# MeDeCom: discovery and quantification of latent components of heterogeneous methylomes

**DOI:** 10.1186/s13059-017-1182-6

**Published:** 2017-03-24

**Authors:** Pavlo Lutsik, Martin Slawski, Gilles Gasparoni, Nikita Vedeneev, Matthias Hein, Jörn Walter

**Affiliations:** 1 Department of EpiGenetics, Saarland University, Campus A2.4, Saarbrücken, 66123 Germany; 2 Machine Learning Group, Saarland University, Campus E1.1, Saarbrücken66123, Germany; 3 Department of Statistics and Biostatistics, Department of Computer Science, Rutgers University, 110 Frelinghuysen Rd, Piscataway, 08854 NJ USA; 4 Present address: Division of Cancer Epigenetics, German Cancer Research Center, Im Neuenheimerfeld 280, Heidelberg, 69120 Germany; 5 Present address: Department of Statistics, Volgenau School of Engineering, George Mason University, 4400 University Drive, MS 4A7 Fairfax, Fairfax, VA 22030-4444 USA

**Keywords:** DNA methylation, DNA methylome, Cell heterogeneity, Deconvolution, Matrix factorization, Epigenetics

## Abstract

It is important for large-scale epigenomic studies to determine and explore the nature of hidden confounding variation, most importantly cell composition. We developed MeDeCom as a novel reference-free computational framework that allows the decomposition of complex DNA methylomes into latent methylation components and their proportions in each sample. MeDeCom is based on constrained non-negative matrix factorization with a new biologically motivated regularization function. It accurately recovers cell-type-specific latent methylation components and their proportions. MeDeCom is a new unsupervised tool for the exploratory study of the major sources of methylation variation, which should lead to a deeper understanding and better biological interpretation.

**Electronic supplementary material:**

The online version of this article (doi:10.1186/s13059-017-1182-6) contains supplementary material, which is available to authorized users.

## Background

DNA methylation is one of the most extensively studied epigenetic marks in the human genome. Methods of detection and quantification are relatively robust and methylation data can be obtained at single-base resolution. DNA methylation closely mirrors the functional state of a cell [1]. Each human cell type has a characteristic methylation profile (methylome) covering its roughly 27 million CpG dinucleotides [2, 3]. DNA methylomes undergo significant global and lineage-related changes during development [4] and form cell-type-specific patterns upon differentiation [3, 5, 6]. They also reflect the individual (genetic) constitution [7], are influenced by gender, are subject to environmental influences [8, 9], and change with age [10]. In aging cells and in diseased cells, they accumulate errors over time and DNA replications [11, 12]. DNA methylation can, therefore, be used to infer the developmental origin, the cell-type specificity, and many other biological and sampling variables contributing to individual epigenetic profiles. A knowledge of these confounding effects and their consequences for methylome changes are of utmost importance for a biological interpretation of DNA-methylation changes in comparative studies.

For practical reasons, comparative epigenomic studies often use tissue samples or cells extracted from body fluids (mostly blood) [3, 13]. All these sources are composed of several major and minor cell types with variable composition [14]. Blood, for example, includes up to ten major and many more minor cell types. Cell type-attributed heterogeneity was shown to be a major source of variation in comparative blood-based DNA methylome studies [15]. The same holds for studies performed with brain tissue, where the compositional changes of cells are strongly influenced by age, gender, and disease state [16–19]. Overall, genetic variation, variable cell composition, and age appear to be the strongest confounders in DNA methylome analysis [20–22].

To overcome the compositional confounding, DNA methylation studies increasingly make use of cell enrichment or cell separation techniques [23, 24] to decompose samples experimentally prior to methylation analysis [25, 26]. These methods clearly enhance the signal interpretability, but they come at the risk of introducing new experimental variation caused by cell-sorting methods, tissue dissection approaches etc. [24, 27]. In the worst case, cell separation may even exclude unknown – but informative – cell populations. Single-cell methylome analyses would be an alternative. However, comprehensive single-cell methylome data are still difficult to obtain and too costly for studies in which large sample numbers have to be compared [28–31]. Moreover, non-uniform cell separation or sampling prior to single-cell approaches may also introduce additional uncontrollable confounding effects. Finally, the sequencing depth has to be high to recover important changes in rare or difficult-to-recover cell populations [31].

Possible approaches for dealing with the heterogeneity problems include computational estimation or correction (adjustment) methods [32]. Houseman et al. were the first to develop a systematic approach that used reference DNA methylation profiles of purified cell types to infer the cell-type proportions in blood via a constrained projection procedure [33–36]. Similar reference-based correction approaches have also been used for complex tissues such as brain [37, 38]. Recently, a series of reference-free methods were developed that adjust for DNA methylation changes caused by cell heterogeneity, allowing for the quantification of direct methylation effects [39–41].

Here we present a novel computational framework called MeDeCom, which uses a special form of regularized non-negative matrix factorization (NMF) to decompose methylome data into a set of underlying latent DNA methylation components (LMCs) and their proportions in each sample. A similar NMF-based approach, RefFreeCellMix, has recently been proposed [42]. However, a key feature distinguishing MeDeCom from RefFreeCellMix and other standard NMF approaches is the incorporation of a biologically motivated regularizer that favors LMCs with per-CpG values close to zero (unmethylated) or one (methylated). In various experiments, we demonstrate that this form of regularization is the key element for an accurate estimation of LMCs corresponding to cell-type-specific methylomes and their associated proportions. Unlike RefFreeCellMix and other NMF-based methods, which infer a correct decomposition only if measurements of pure cell-types are implicitly present in the data set, MeDeCom also works when only measurements of mixtures of different cell types and no purified references are available. We demonstrate the performance of MeDeCom in controlled experimental settings and its application in more complex scenarios of cell populations and tissues. We show that MeDeCom can be used for adjustment in an epigenome-wide association study (EWAS) with excellent performance on par with the most advanced methods [39–41]. Finally, we demonstrate that the unsupervised decomposition of complex methylation data into LMCs and their proportions can be used as a new exploratory tool to obtain novel biological insights going beyond the analysis of confounding factors.

## Results and discussion

### MeDeCom: introduction to the computational framework

**Fig. 1:**
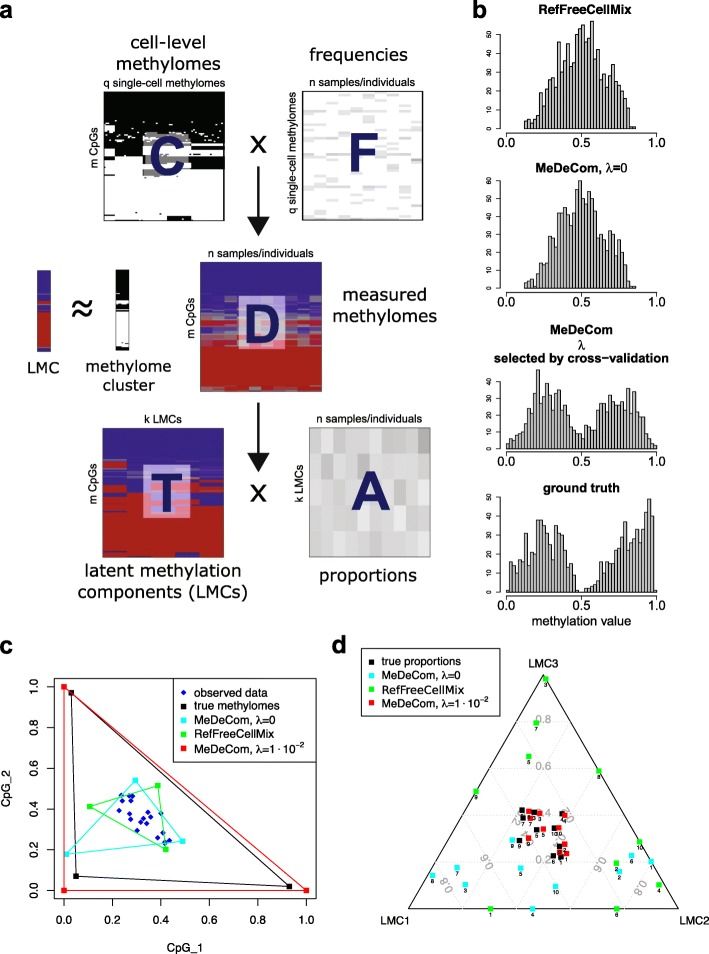
Computational framework of MeDeCom. **a** The conceptional background of MeDeCom. The measured methylomes (e.g., as 450K data, shown in the center) can be seen as a composition of binary single-cell methylome signatures (*C*) with their frequencies in each sample (*F*). Single-cell signatures of a particular cell type form a cell-type specific cluster in *C*. MeDeCom decomposes the measured methylation data into a matrix *T*, representing latent methylation components (LMCs), which in turn correspond to the averaged cell methylomes of a cell-type-specific cluster in *C*, and into *A*, the relative proportions of LMCs (respectively, cell types) in the sample. **b** Histograms of the values in the estimated *T* matrices for the 500 most varying CpG sites for the cell reconstruction experiment of neuronal cells (see text). We observe that both MeDeCom with no regularization (*λ*=0), and RefFreeCellMix are unable to match the distribution of the reference profiles (ground truth), which is biased towards zero and one. However, MeDeCom with our regularizer (parameter *λ* is chosen by cross-validation) biases the entries of the LMCs towards zero (unmethylated) and one (methylated). Thus, the distribution of the entries of the estimated LMCs matches approximately the ground truth leading to a significantly better estimation of *T* as well as *A*. **c**-**d** Geometric intuition about the different methods for a fully synthetic example of two CpGs (*n*=30, *k*=3). Each LMC corresponds to a column of *T* and, thus, is a point in [0,1]^2^. **c** shows the estimated LMCs (*squares*) of RefFreeCellMix and MeDeCom with *λ*=0 and *λ*=10^−2^, and the ground truth (*black squares*) together with the data (*blue dots*). The data points are mixtures of the ground truth points and, thus, lie in the convex hull of the latter. Factorization problem (2) (see “Methods”) is ill-posed as the solution is not unique. MeDeCom with appropriate regularization estimates *T* (*red squares*) very accurately as the solution is biased towards zero or one, whereas RefFreeCellMix and MeDeCom with *λ*=0 are unable to find the correct LMCs. This also leads to huge errors in the estimation of the proportions as visualized by the ternary plot for ten randomly selected data points (**d**). In contrast, MeDeCom with appropriate regularization estimates *A* very accurately

We developed MeDeCom, a novel computational framework for methylation data decomposition. The conceptual background of MeDeCom is illustrated in Fig. 1 a. DNA methylation profiles of complex tissues and cell mixtures are a composite mix of patterns of individual cell types with discrete (binary) position-specific methylation values (Fig. 1 a). In other words, the DNA-methylation pattern generated, e.g., using 450K or EPIC bead arrays, is the product of the cell-specific pattern variation *C* and the frequencies in which individual cells are present in tissues or cell mixes, *F* (Fig. 1 a, top). MeDeCom decomposes such mixed methylome patterns into two matrices, *T* and *A*. *T* describes the LMCs and reflects an average methylation pattern of an underlying cell type, while *A* contains the proportions of LMCs in each sample (Fig. 1 a, bottom).

To estimate *T* and proportions *A* for LMCs, MeDeCom uses a constrained NMF algorithm together with a regularization function on *T*. The regularization shifts the estimated matrix of methylation patterns *T* towards biologically plausible binary values close to zero (unmethylated) or one (methylated). The regularization of *T* is key to yielding accurate estimates of cell-type-specific methylation patterns and their proportions (see below). MeDeCom has two parameters: i) the number *k* of LMCs that are supposed to be estimated and ii) the amount of regularization *λ*. We show that both parameters can be reliably estimated by cross-validation. The details and the mathematical background of MeDeCom are outlined in “Methods.”

To facilitate the interpretation of the MeDeCom results, we designed an exploratory interactive visualization tool called FactorViz. This tool allows the user to visualize the performance of MeDeCom, explore the LMCs, and obtain various kinds of information for further biological interpretation. MeDeCom and FactorViz are publicly available as a web resource at [43].

In the following sections, we will demonstrate the use of MeDeCom on synthetic and real Infinium 450k data sets of increasing complexity. We also demonstrate the usefulness of MeDeCom to decompose complex blood and tissue methylation data (also in comparison to reference-based methods) and provide examples showing how the obtained LMCs can help explore the origin of variation. We will adjust these parameters and provide novel ideas for the biological interpretation of methylation data.

#### Illustration of the effect of regularization

While conceptually simple, the introduction of our biologically motivated regularizer is the major determinant of the superior decomposition achieved by MeDeCom (Fig. 1 b). The histograms of the estimated *T* matrices are shown for an unregularized model and the regularized model chosen by cross-validation (a more detailed description of the corresponding cell reconstruction experiment follows below). The histogram of *T* for the regularized model is very close to the histogram of the true methylomes, while the histograms of the unregularized model and of RefFreeCellMix are far from the ground truth, which reflect the lack of bias towards biologically plausible *T*. The correct estimation of *T* via regularization allows us also to recover the correct proportions (Fig. 1 c, d). In our model scenario, all data points (blue dots) lie in the convex hull of the three estimated LMCs (squares), showing that there exist multiple solutions with virtually the same fit to the data. MeDeCom breaks this ambiguity in the solution as the regularizer shifts the values of the LMCs towards zero and one. We see that the regularized model fits the ground truth well (Fig. 1 c). A misestimation of *T* also leads to a misestimation of the proportions in *A* (Fig. 1 d). The proportions of the three LMCs in each sample as estimated by MeDeCom are very close to the true ones for the regularized model while they are completely wrong for the unregularized model and RefFreeCellMix.

#### Decomposition of simulated methylation data

To examine the performance of MeDeCom in a controlled setting, we analyzed synthetic DNA methylation mixtures generated by simulation (see “Methods” for details). The controlled data sets varied in the numbers of cell-type-specific patterns (LMCs), the inter-LMC similarity, and the variability of the mixture proportions (see Additional file 1: Table S1).

**Fig. 2:**
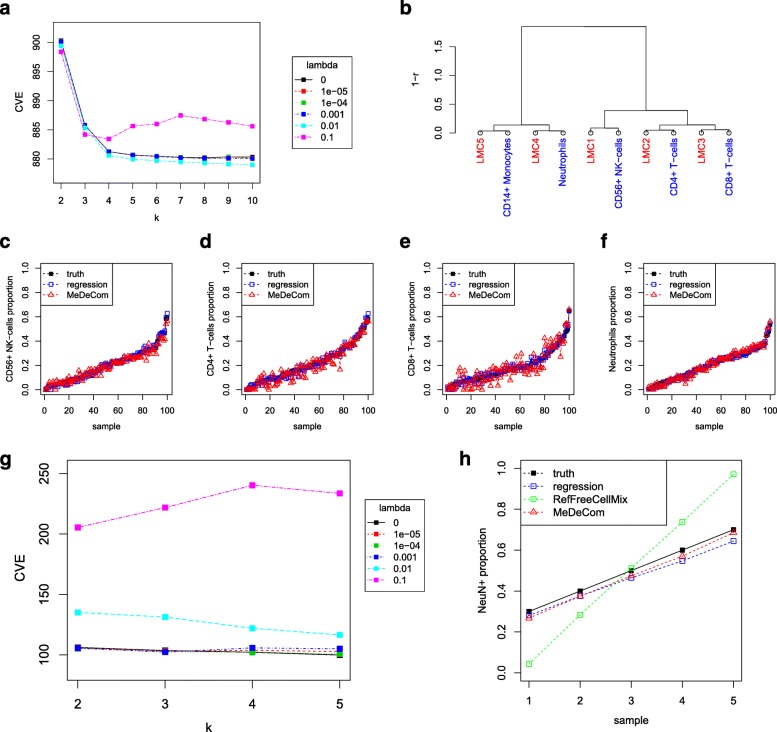
Testing MeDeCom on simulated and artificial cell mixture data. **a**–**f** Results for the simulated data example with five methylation components, moderately variable mixing proportions, and medium noise level. **a** Selection of parameters *k* and *λ* by cross-validation. **b** Matching of the recovered LMCs to the true underlying profiles. The dendrogram visualizes the agglomerative hierarchical clustering analysis with correlation-based distance measure and average linkage. **c**–**f** Recovery of the mixing proportions. *Truth* stands for true mixing proportions and *regression* denotes the reference-based proportion estimation as described in “Methods.” In each line plot, the synthetic samples are sorted by ascending true mixing proportion. **g**, **h** Results for the ArtMixN data set. **g** Selection of parameters *k* and *λ* by cross-validation. **h** Recovery of mixing proportions (only NeuN ^+^ is shown) for MeDeCom and RefFreeCellMix. RefFreeCellMix misinterprets the most extreme mixtures as pure cell types and, thus, estimates *T* (see Fig. 1b) as well as the proportions in *A* wrongly. Notation is the same as in **c**–**f**

Figure 2 a–f summarizes the results for moderately variable mixture proportions of five pure blood-derived cell-type profiles (see below). FactorViz inspections show that the cross-validation error (CVE) levels out at *k*≥5, indicating that MeDeCom identified the correct number of underlying LMCs (Fig. 2 a). The optimal regularization parameter *λ* was found to be *λ*=0.01. The estimated LMCs unambiguously match the source DNA methylation profiles (Fig. 2 b). The individual methylation profiles were reconstructed with an overall root-mean-square error (RMSE) of 0.064. MeDeCom also accurately reproduced the mixing coefficients (proportions) with mean absolute error (MAE) of 0.0296 (Fig. 2 c–f). We obtained similar results for other cases with a varying number of underlying components and mixture proportions (see the MeDeCom web resource).

The summary plots of the LMC recovery rate (Additional file 2: Figure S1) show that, given a low number of samples, the choice of the model and the variability level of the mixture proportions were key factors in the performance of LMC reconstruction in MeDeCom. However, decomposition became impossible when the variability of the mixture proportions was very low and, at the same time, the noise level was high (see an example in Additional file 2: Figure S2 and the MeDeCom web resource). In this case, the variation in the data due to uneven cell-type composition is comparable or smaller than the noise, and, thus, it becomes impossible to estimate LMCs and their proportions. We also did the same experiments for RefFreeCellMix. For simple cases, it performs similarly to MeDeCom, but RefFreeCellMix is outperformed consistently by MeDeCom once the setting gets more difficult (Additional file 2: Figure S1).

#### Decomposition of reconstructed cell mixtures

Next, we analyzed the performance of MeDeCom on publicly available 450K data sets of cell mixtures with known proportions [37] (data set ArtMixN in Table 1). In this study, brain cell nuclei were separated using a neuron-specific marker NeuN, and fluorescence activated cell sorting (FACS) into NeuN ^+^ (neuronal) and NeuN ^−^ (non-neuronal) fractions. These fractions were mixed incrementally (Additional file 1: Table S2) and methylomes measured on a 450K array. We were interested in finding out how well MeDeCom could recover the source NeuN ^+/−^ methylomes and their mixing ratios. We show the results for five mixtures: {(0.3,0.7),(0.4,0.6),(0.5,0.5), (0.6,0.4),(0.7,0.3)}. The results for all nine mixtures can be found in Additional file 2: Figures S6 and S7.

**Table 1:** Public Infinium 450k data sets used in the study

ID	Source	GEO Accession	Brief description	*n*	Reference
*Blood data sets*					
PureBC	[44]	GSE35069	Seven MACS-purified blood cell types from blood of six healthy male donors: neutrophils, monocytes, B cells, CD4+ and CD8+ T cells, NK cells, and eosinophils	42	
WB1	[35]	GSE42861	Whole blood of healthy controls from a rheumatoid arthritis study (technical batch II)	87	PureBC
WB2	[45]	GSE51032	Whole blood of the EPIC Italy study participants who remained cancer-free in 2010	442	PureBC
*Neuronal data sets*					
PureN	[37]	GSE15745	Cortical NeuN ^+/−^ fractions of the 29 healthy controls	58	
ArtMixN	[37]	GSE15745	Nine titration mixtures of the NeuN ^+/−^ fractions	9	PureN
FC1	[37]	GSE15745	Frontal cortex of ten MDD patients and ten healthy controls	20	PureN
FC2	[19]	GSE15745	Frontal cortex from a large AD study	114	PureN

*AD* Alzheimer’s disease, *GEO* Gene Expression Omnibus, *MDD* major depression disorder

MeDeCom indeed identified two major LMCs at CVE minimum close to *λ*=5×10^−4^ (Fig. 2 g; Additional file 2: Figure S3). Each of the recovered LMCs showed high CpG-wise correlation to the average profile of either the NeuN ^+^ or NeuN ^−^ fractions (Additional file 2: Figure S4) and reproduced it with high accuracy (RMSE 0.029). The mixture proportions were accurately recovered as well (MAE 0.025; Fig. 2 h). As in the artificial example of Fig. 1 c, RefFreeCellMix is inferior both in the estimation of *T* (RMSE 0.037) and *A* (MAE 0.162) due to the lack of a bias towards biologically plausible values (Fig. 2 h and Additional file 2: Figure S5). The difference in the results for MeDeCom and RefFreeCellMix becomes even more pronounced if one computes the RMSE for *T* limited to the 500 most varying CpG sites, where MeDeCom has a RMSE of 0.082 compared to 0.190 for RefFreeCellMix and 0.194 for MeDeCom with no regularizer (*λ*=0). In Fig. 1 b, we visualize the difference by showing the histogram of the estimated entries of *T* for the 500 most varying CpG sites. Our estimated histogram is close to the ground truth whereas the histograms of RefFreeCellMix and the unregularized model are much further off, which is then reflected in the wrong estimation of the proportions for RefFreeCellMix. Since the synthetic experiments as well as the artificial mixture experiment show that RefFreeCellMix cannot reliably recover cell-type LMCs and their proportions when there are only mixtures as samples, we do not compare to them in the analysis of complex mixtures from blood or brain tissue.

### Methylome decomposition of whole-blood cell samples

Following the successful test of MeDeCom on synthetic data and artificial cell mixtures, we applied our method to whole-blood Infinium 450k samples from two independent studies (Table 1). Our aim was to test the performance, reproducibility, and robustness of our method in a side-by-side comparison. We first applied MeDeCom to control samples from a large rheumatoid arthritis study [35]. To avoid known technical confounding effects (Additional file 2: Figure S9), we confined our first analysis to 87 samples forming a technically homogeneous batch (data set WB1).

**Fig. 3:**
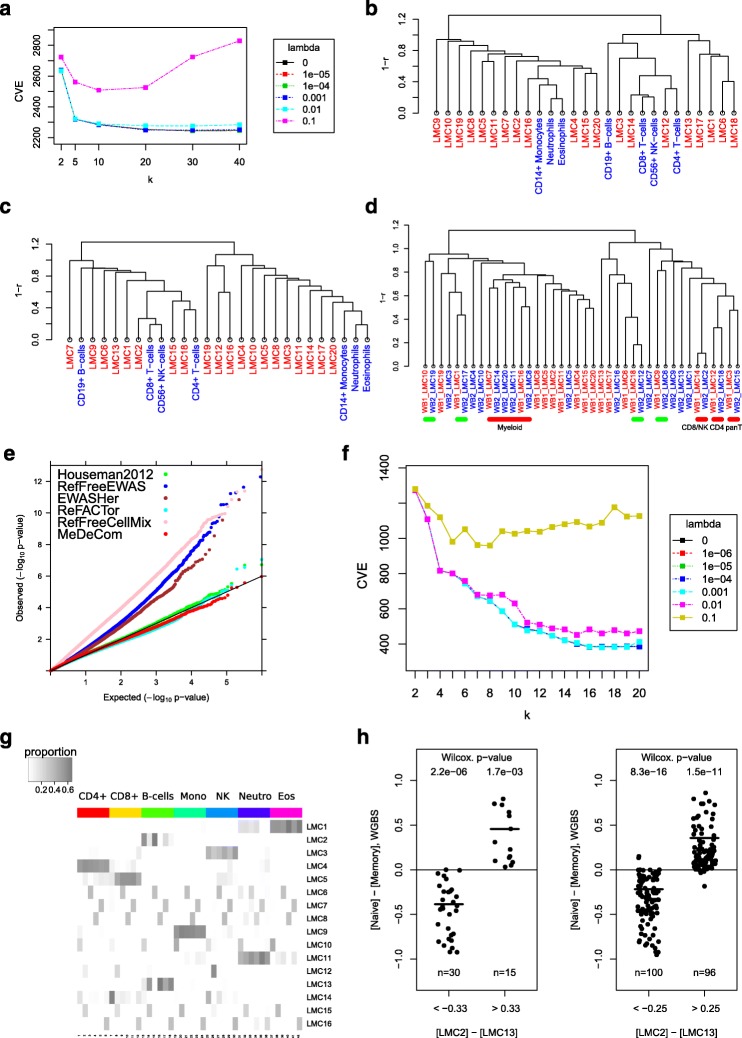
Results for blood cell methylomes. **a**–**e** WB1 data set. **a** Selection of parameters *k* and *λ* by cross-validation. **b** Matching the WB1 LMCs to PureBC methylomes (*k*=20, *λ*=0.001). Here and below the dendrogram visualizes agglomerative hierarchical clustering analysis with a correlation-based distance measure and average linkage. **c** Matching the LMCs from the WB2 data set (*k*=20, *λ*=0.001) to the PureBC methylomes. **d** Matching the WB1 and WB2 LMCs to each other. Pairs of reproducible LMCs also matching to the reference profiles are highlighted by *red segments*. *Green segments* mark reproducible LMCs that do not directly match any of the reference profiles. **e** Adjustment of the association analysis for rheumatoid arthritis in the full Liu et al. data set [35]. Each curve is a Q-Q plot of *P* values observed in the corresponding analysis versus the expected *P* values sampled from a uniform distribution. **f**–**h** PureBC data. **f** Selection of parameters *k* and *λ* by cross-validation. **g** Heat map of recovered proportions in PureBC data (*k*=15, *λ*=0.001). Rows represent LMCs while columns correspond to individual purified samples. The order of blood donors is the same within column sets, corresponding to one cell type. **h** Methylation differences in naive versus memory B cells at CpGs differentially methylated between LMC2 and LMC13 from the PureBC data set. WGBS methylation profiles of naive and memory B cells were obtained from BLUEPRINT. The value for memory B cells is an average of three WGBS samples. A Wilcoxon ranked sum test was used to test the null hypothesis that WBGS methylation calls are the same in naive and memory cells at their respective CpG positions

The CVE continued to decline until *k*=20 implying a large number of distinct variation confounders that is, LMCs (Fig. 3 a). We, therefore, examined the factorization results for increasing values of *k* to understand the relation between LMC recovery and underlying major and minor confounding variants (i.e., cell types, subtypes etc.). For a biological interpretation, we compared the LMCs of increasing values of *k* to published reference methylomes of FACS-sorted major blood cell types [44] (data set PureBC).

From *k*=2 on, the recovered LMCs distinguish the cell populations of the myeloid and the lymphoid lineages, respectively (Additional file 2: Figures S10 and S11). This split in the two lineage clusters is maintained at increasing values, e.g., *k*=20, *λ*=1.0×10^−3^ (Fig. 3 b; Additional file 2: Figure S12). Altogether, 11 LMCs in the myeloid arm cluster show greater similarity to LMCs describing FACS-sorted references for monocytes, eosinophils, and neutrophils while the remaining nine LMCs cluster with CD4+ T cells, CD8+ T cells, NK cells, and B cells. In the myeloid cluster, we fail to detect direct sub-lineage-specific LMC matches. In the lymphoid cluster, however, we observed one LMC closely matching the CD4+ T-cell profile, and one LMC corresponding to the sub-cluster of CD8+ T cells and NK cells, indicating a better separability of the T-cell signatures based on the 450K data used. Finally, our analysis directly identified a number of LMCs with high proportions in single donors, most probably reflecting sites with genetic variation (Additional file 2: Figures S13 and S14).

The results of the first data set were reproduced on a second independent whole-blood data set (WB2) from the EPIC Italy study [45], which recovered a highly similar clustering of LMCs (Fig. 3 b and c). A direct comparison of the LMC clustering between both whole-blood data sets reveals a considerable agreement of LMCs matching side by side, suggesting that MeDeCom recovers robust and reproducible LMC signatures (Fig. 3 d).

An aggregated comparison of LMCs matching to reference cell types in both blood analyses showed good correspondence to the regression-based estimations of cell proportions (Additional file 2: Figure S15). For several LMCs in WB1 and WB2, we observed that their proportions correlated with age, e.g., for WB1 the LMC12 related to CD4+ T cells (Additional file 2: Figure S17). Although the total number of CD4+ T cells was reported to change non-significantly with age [46], T-cell-specific immunological senescence is a well-known phenomenon characterized by depletion of the naive T-cell sub-populations [47, 48]. This might imply that LMC12 rather reflects the methylation pattern of the naive CD4+ T cells. Indeed, a comparison to reference methylomes of isolated T cells supports this suggestion (Additional file 2: Figure S16).

#### Correction of the phenotype association analysis

Next, we examined if LMCs estimated by MeDeCom can be efficiently used for data adjustment in phenotype association analyses. We first verified the potential of the adjustment in a fully synthetic setting mimicking a typical EWAS in blood (see “Methods” for details). We added true methylation effects at the level of single cell type as well as confounding by cell-type proportions (Additional file 2: Figure S29a and b). Adjustment for LMC proportions indeed helped to decrease the confounding and recover the true methylation effects with performance close to the reference-based adjustment of the best available third-party methods (Additional file 2: Figure S29c–e).

We then applied this approach to a large rheumatoid arthritis data set (WB1), which previously has been used by others for confounding corrections [35, 39–41]. We started by selected CpGs significantly associated with the rheumatoid arthritis status using linear modeling (see “Methods” for details) with and without adjustment for common covariates. The deviations of the observed *P* value distribution from the expected uniform distribution indicated a large inflation of significance (Additional file 2: Figure S19a). This effect is due to the confounding caused by the unequal distribution of the cell types in rheumatoid arthritis patients and controls [35]. We then performed an independent correction for cell composition variability using one reference-based [33] and four reference-free methods [39–42], and compared these results to the results obtained with MeDeCom. Comparative Q-Q plots of the *P* values show that the methods indeed decrease the inflation of significance (Fig. 3 e and Additional file 2: Figure S19). In this test, the adjustment using the LMCs estimated by MeDeCom showed comparable performance with reference-based analysis and the results of ReFACTor. We conclude that the LMCs generated by MeDeCom are useful for covariate correction.

#### Purified blood cell populations

Our whole-blood analyses revealed a limitation in an unambiguous assignment of reference cell types by single LMCs, which may have several reasons. One possible explanation is that the methylomes of FACS-sorted CD marker-positive purified cells, which we and others use as references, constitute composed methylomes of donors with a varying content of cell subtypes. First, a recent single-cell-resolution study of transcriptional heterogeneity in mammalian hematopoiesis [49] revealed that the potential of the canonical cell-surface markers to discriminate fine blood cell populations is limited, and their use as FACS gates for cell separation is prone to errors. Second, in particular for B and T cells, it is known that the proportion of cell subtypes may vary and different types of quiescent or dividing cells, such as naive, effector, or memory sub-populations, may confound a clear LMC assignment. We addressed this question by performing a MeDeCom analysis on the seven purified blood cell populations derived from six donors (data set PureBC) [44].

In this analysis, the CVE stabilized at *k*=16 and *λ*=10^−3^ (Fig. 3 f; Additional file 2: Figures S21 and S22). A matrix of mixture proportions (Fig. 3 g) showed that the recovered 16 LMCs could be classified into two distinct groups. Six LMCs (LMCs 6, 7, 8, 10, 15, and 16) could be associated with individual donors, most likely reflecting donor-specific genetic variation at the informative CpG positions underlying these LMCs. In a second group, LMCs 1, 3, 4, 5, 7, 9, and 11 corresponded to the enriched cell-type-associated profiles; e.g., LMC4 was predominantly present in CD4+ T cells, LMC11 in neutrophils etc. Nevertheless, we also observed that several LMCs were shared by related cell types. For instance, eosinophil samples show enrichment of the neutrophil-specific LMC11, CD8+ T cells (LMC9) show overlaps with CD4+ T cells (LMC5). Finally, we observed LMCs that were associated with more than one cell type, but which were not a dominating LMC in any of them. For instance, LMC14 was present at low proportion both in CD8+ T cells and NK cells. The co-occurrence of two or more LMCs within one isolated cell population, as well as sharing of LMCs between the populations, suggests that these cell populations could be either mixtures of still not separated distinct cell types, or that these cell populations share epigenetic features that may indeed co-occur in different cell types.

A clear split for sub-population heterogeneity was observed for CD19+ B cells. Here two LMCs, LMC2 and LMC13, apparently separate naive and memory B cells. To support this conclusion, we selected 401 CpG positions with a methylation difference of more than 0.33 in LMC2 compared to LMC13. First, we saw that many of these CpGs were located in the vicinity of known B-cell-associated genes (Additional file 3), such as *PTPRCAP* (Additional file 2: Figure S23). We then compared the LMC2- and LMC13-specific CpG 450K values to reference WGBS methylome profiles of memory and naive B-cell samples, obtained by the BLUEPRINT project [50]. Then, 44 CpGs (Additional file 3) indeed directly correspond to the methylation state differences reported by Kulis et al. [50] in memory and naive B-cell sub-populations, respectively (Fig. 3 d). We would like to note that LMC2 and and LMC13 have almost inverse proportions for individual donors, indicating that the MeDeCom analysis directly reflects the differences in sample-specific abundance of memory and naive B cells, which suggests individual- or isolation-attributed variation.

In our blood analysis, we observed that CpGs, which clearly discriminated cell types in purified myeloid and lymphoid lineages, did not exhibit this power in complex samples. To understand this better, we preselected 15,000 marker CpGs with the highest discriminative power between cell types (highest CpG-wise *p*=2.91×10^−44^, ANOVA F-test). A visual comparison of these CpGs between individual reference populations and whole-blood data (Additional file 2: Figure S18) clearly showed that they have a rather low variation across whole-blood samples. Indeed, only a relatively small proportion of marker CpGs also showed a high variance across whole-blood samples detectable by MeDeCom (see the row color code in Additional file 2: Figure S18). We conclude that CpGs, which can be assigned to isolated cell types in purified myeloid and lymphoid lineages, are less informative in complex samples since their level of informative variation in an NMF-based analysis of whole blood is low. This may be a second reason to explain why a series of LMCs recovered in whole blood and in the extreme cases of our simulations do not unambiguously match the reference methylomes.

### Decomposition of the brain tissue methylomes

Next we applied MeDeCom to examine the heterogeneity of tissue methylomes. The human brain is composed of many neuronal and glial cell types. Current studies apply FACS-based methods to separate glial cells and neurons. The RBFOX3 protein localized in the nuclear membrane of most neuronal cells (also known as NeuN) is used as a selection marker. While the NeuN-enriched and NeuN-depleted cell fractions serve as references in methylome analysis, the question remains to which extent these separated methylomes represent the composition of whole-brain tissue.

We applied MeDeCom to 20 frontal cortex methylomes from a major depression disorder study [37] (data set FC1 in Table 1). The data set also included NeuN ^+^ and NeuN ^−^ cell fractions (data set PureN), which we analyzed in comparison to total brain tissue. In addition, we examined an independent bulk frontal cortex methylome data set from a recent large-scale Alzheimer’s disease (AD) study [19] (data set FC2).

**Fig. 4:**
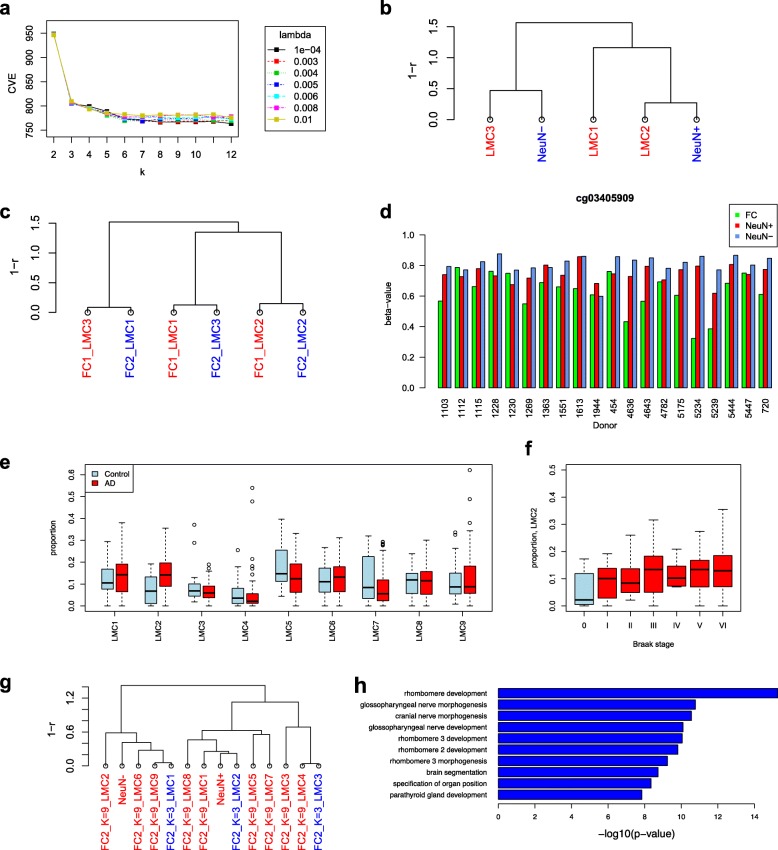
Results for brain methylomes. **a**–**d** Decomposition of the FC1 data set. **a** Selection of parameters *k* and *λ* by cross-validation. **b** Matching frontal cortex LMCs to the reference NeuN ^+/−^ profiles. The dendrogram visualizes agglomerative hierarchical clustering analysis with a correlation-based distance measure and average linkage. **c** Matching of LMCs between FC1 and FC2. **d** Example of an LMC1-specific CpG (*k*=3) in the PAX6 locus. **e**, **f** AD-associated LMCs in the FC2 data set. **e** LMC2 is associated with the AD phenotype (Wilcoxon rank sum test *P*=3.1×10^−4^). **f** LMC2 is also significantly associated with the Braak stage (*P*=4.8×10^−3^, *T* test of the linear regression coefficient). **g** Clustering of the recovered LMCs for *k*=9 with the LMCs for *k*=3 and reference profiles. LMC2 belongs to the NeuN ^−^-associated cluster. **h** Most significant gene ontology terms from the biological process category for the LMC2-associated hypermethylated genes

For both the FC1 and FC2 data sets, the inspection of CVEs showed a substantial change at *k*≥3, strongly suggesting the existence of more than three main epigenetically distinct cell components (LMCs) (Fig. 4 a and Additional file 2: Figure S24). We carefully examined the factorization results and compared the three main LMCs at *k*=3 and *λ*=5×10^−3^ to the NeuN ^+^ and NeuN ^−^ profiles. Clustering analysis (Fig. 4 b) showed that the average NeuN ^−^ reference profile is related to LMC3 while the NeuN ^+^ profile is more similar to LMC2. The third component LMC1 was truly distinct from both reference methylomes retaining a slightly higher similarity to LMC2 and the NeuN ^+^ methylome. All three LMCs were remarkably well reproduced in the independent FC2 data set at *k*=3 (Fig. 4 c).

This finding indicates that the FACS separation of brain tissues into NeuN ^+^ and NeuN ^−^ cells introduces a new confounding variable. In most cases, the NeuN ^+^ and NeuN ^−^ fractions together do not fully recapitulate the methylomes of total brain tissues. To get more insights into the biological nature of the LMCs, we asked which loci differ in their methylation between the LMCs and examined the biological annotation of genes associated with LMC-specific CpGs. LMC-specific CpGs were selected to have methylation differences more than 0.33 between one LMC against the two other ones (Additional file 4). We then mapped LMC-specific CpG positions to their neighboring genes (Additional file 4; see “Methods”) and performed a functional annotation of the associated genes using GREAT [51] (Additional file 2: Figure S25). LMC2 (NeuN ^+^)-specific CpGs map to genes with a clear enrichment for neuronal-related terms, while LMC3 (NeuN ^−^)-specific CpGs were close to genes associated with non-neuronal, mostly oligodendrocyte-related, categories. LMC1-specific CpGs map close to genes associated with developmental and stem-cell-related terms. Strikingly, among the genes associated with LMC1, we found several markers of the neuronal stem-cell lineage, such as *PAX6*, *ZIC1*, *ZIC4*, and *NEUROG1* (Additional file 4). Notably, the DNA methylation patterns at LMC1-specific CpGs showed significantly higher or lower methylation levels in crude brain tissue than in NeuN ^+^ and NeuN ^+^ reference methylomes (see *PAX6* as an example in Fig. 4 d and Additional file 2: Figure S26). Furthermore, a recent study on neuronal heterogeneity in the mouse brain [52] provided a reference for the fine cellular subtypes possibly present in the mammalian frontal cortex. We found several of the most significant LMC1-specific genes among the DMRs reported in [52] (Additional file 1: Table S3).

As outlined earlier, LMC proportions tended to be biased when *k* was significantly lower than optimal (see the WB1 analysis with *k*=2 above). We, therefore, explored MeDeCom results at *k*=4 and *λ*=0.005 (Additional file 2: Figure S27). The analysis revealed that the NeuN ^+^-specific LMC3 rather accurately reproduced a reference-estimated NeuN ^+^ content in most brain samples (Additional file 2: Figures S7a and S28a). However, samples with the highest deviation from the reference-based proportions had the highest proportion of co-purified cells (and methylomes), characteristic of LMC2 (equivalent to LMC1 for *k*=3; Additional file 2: Figures S27b and S28b). For *k*=4, two LMCs match to NeuN ^−^. For each of them, the proportions recovered by MeDeCom deviated significantly from the reference-based estimates for NeuN ^−^ (Additional file 2: Figure S28c and d). Nevertheless, the combined proportions largely reflected the reference-estimated NeuN ^−^ content across all samples (Additional file 2: Figure S28e). Again, we observe that samples with the lowest correspondence had a high contribution of LMC2 (Additional file 2: Figure S28f). The proportion analysis shows that by using MeDeCom we can infer realistic LMC proportions for NeuN ^+^, NeuN ^−^ in individual samples, and a third separate LMC with a distinct cell composition. The latter LMC is variably convoluted into the other main NeuN ^+^ and NeuN ^−^ cell fractions in the reference-based analysis. We conclude that reference-independent decomposition is a very helpful approach for exploring, identifying, and quantifying heterogeneity effects across composite tissue samples and will allow us to obtain important and unbiased correction parameters for epigenetic studies of the brain.

### Discovery and annotation of the AD-related LMCs

Finally, we applied MeDeCom for a phenotype-related analysis on samples where reference methylome adjustment is impossible. To demonstrate the exploratory potential of MeDeCom over other methods in such a setting, we first tested MeDeCom on a simulated data set with an admixture of a rare cell population in one of the compared sample groups as the only phenotype-related effect (see “Methods”). In this example, MeDeCom correctly estimated the number of underlying methylation components, and revealed the enrichment of the rare LMC only in the case group (Additional file 2: Figure S30). Encouraged by these results, we applied MeDeCom for an association analysis of the AD phenotype in the FC2 data set. The authors used a canonical CpG-wise approach to identify methylation changes associated with AD. Braak staging was used as a main phenotypic readout. Standard linear modeling using Braak stage as the response variable corrected for sex and age at death revealed residual inflation of significance, arguing for the presence of an unknown confounding variability component (Additional file 2: Figure S31a). A search for the strongest associations with LMC proportions across all obtained factorization solutions revealed that for the decomposition case with *k*=9 and *λ*=0.09, the proportion of some LMCs, in particular LMC2, is significantly correlated with both AD phenotype and Braak stage (Fig. 4 e and f). When we included the proportions of the three most significant LMCs as covariates in the association analysis, the remaining *P* value inflation was eliminated (Additional file 2: Figure S31b). When compared to the LMCs recovered at *k*=3, LMC2 was the closest to the NeuN ^−^-related cluster (Fig. 4 g). We used GREAT to annotate the LMC2-specific CpG positions. Gene ontology terms with significant enrichment included rhombomere development, brain segmentation, nerve morphogenesis etc. (Fig. 4 h). We also observed an enrichment for gene promoters overlapping the vitamin D receptor and MEIS1 binding motifs (Additional file 2: Figure S32). LMC2 might, therefore, represent one or several cell populations that are enriched in AD samples; however, a more in-depth biological analysis and validation would be necessary to confirm this finding.

## Conclusions

DNA methylomes of multicellular samples can be modeled as mixtures of several latent variables. Here we present a novel computational framework called MeDeCom, which decomposes complex DNA methylation data into latent components and sample-dependent proportions based on a mixture model for methylomes. We show that the method performs reproducibly and with high sensitivity on both synthetic and biological data sets.

MeDeCom provides significant advances compared to existing methods. First of all, our method does not require reference cell-type measurements. It can be applied to any DNA methylation data set to explore the compositions of mixtures. Note that reference methylome data are not yet available for many cell types, and MeDeCom offers the possibility of exploring non-standard data in a reference-free manner. Second, MeDeCom has strong conceptual differences to other reference-free methods, such as the surrogate variable analysis (SVA) methods [53, 54], EWASHER [40], or the SVA-inspired RefFreeEWAS [39] method. All these methods focus on the correction of significance analysis for a phenotypic trait of interest by calculating and eliminating confounding heterogeneity effects. In contrast, MeDeCom uses a variant of NMF specifically designed to recover latent DNA methylomes by using biologically motivated constraints and regularization. The imposed constraints on the factorization integrate biological prior knowledge, such as non-negativity of the estimated methylation profiles and their proportions. However, we show that these constraints alone are not sufficient to get biologically meaningful methylation profiles and accurate estimates of their proportions. A key element distinguishing MeDeCom from other methods based on naive matrix factorization, in particular RefFreeCellMix [42], is that we add a regularizer encoding the prior expectation that most sites in the methylation profiles are close to zero or one. This prior expectation is because at the level of a single cell, methylation profiles are binary and for most CpG sites this is true also at the level of a homogeneous population of cells, such as a particular cell type. This allows us to estimate methylation profiles and their proportions simultaneously, without any reference profiles. In contrast to RefFreeCellMix, the employed regularizer enables MeDeCom to identify methylation profiles even in blood and brain tissue where each sample is a heterogeneous mixture of different cell types.

Our proof-of-concept analysis shows that MeDeCom acts robustly and reliably on complex artificial and natural methylome mixtures measured by Infinium 450k arrays. MeDeCom identifies key signatures of major cell populations present in complex whole-blood and brain methylomes without any prior knowledge of references or data adjustment. However, our analysis also reveals the limits of a MeDeCom analysis. The method strongly depends on a fair number of discriminatory methylation positions and a sufficient level of sample-to-sample variation (Additional file 2: Figure S18). In complex 450k whole-blood methylomes, both parameters are affected such that a clean separation and assignment of LMCs specific for blood cell subtypes becomes challenging. Two major aspects are the likely causes of this difficulty. First, the Infinium 450k platform covers only a limited number of CpGs informative for the minor cell subtypes, which can easily become indistinguishable from the remaining technical noise of the 450k arrays. Second, the proportions of most cell subtypes in blood are too low. MeDeCom factorization requires a certain grade of sample-to-sample variation to identify component (cell type)-specific CpG signals. We had noticed both of these limitations in our simulation analysis with artificial mixtures. In the future, these problems may be partially overcome by using WGBS/RRBS or extended array platforms such as the Methylation EPIC array covering additional cell-type-specific variable CpG positions. Furthermore, cell-enrichment or cell-depletion strategies may help to obtain deeper sample-specific compositional insights.

Since MeDeCom does not require predefined references, it can be flexibly applied to any level of methylome analysis. We show that MeDeCom can facilitate a deeper insight into cell composition if the sample complexity is experimentally reduced. As one example, we investigated the composition of methylomes generated after cell preselection, e.g., by surface marker-based separation [44]. Our results on pre-sorted CD4+ (T cell) or CD19+ (B cell) blood cells clearly show that their methylomes still maintain a substantial level of heterogeneity. We identify a number of additional separable DNA methylation components, some of which we can associate with age-dependent changes in T-cell populations or show that they discriminate naive from primed B cells. In both cases, the characteristic CpG signatures vary in their sample-by-sample proportions. Such observations are very important for the biological interpretation of methylation changes across populations of samples. Many of the components identified by MeDeCom are likely to carry such biological information, which can be extracted for further exploration. Furthermore, we show that MeDeCom can, in principle, be extended to include prior information, such as known cell-type profiles and the approximate range of cell-type proportions for certain cell types (Additional file 5: Supplementary Note 2).

The decomposition of brain methylomes provided by MeDeCom further supports the usefulness of unsupervised exploratory decomposition for the analysis of complex methylome data. The separation of brain cells into neuronal and non-neuronal fractions has become a new standard procedure for brain-specific epigenetic studies in human postmortem samples. Our first finding shows that NeuN ^+/−^ mixture models do not fully capture the composition of the full brain tissue. In fact, we identify an additional component that differs from the NeuN ^+^ (neuron) and NeuN ^−^ (non-neuron)-specific components in full brain tissues. This new component is apparently sorted out or even lost in the enrichment procedure. Our analysis shows that the samples denominated as NeuN ^+^ and NeuN ^−^ contain variable contributions of this unknown cell fraction. Here, MeDeCom opens a new possibility for identifying the differences in cell composition and, hence, making data from different NeuN separations more comparable. Moreover, a biological analysis of the CpGs and genes associated with this new component reveals a strikingly different association of biological terms compared to the NeuN ^+^ and NeuN ^−^ fractions. Finally, we show that a phenotypic re-analysis of complex brain data sets using LMCs allows us to identify novel associations with cellular origin (neurons) and disease-state progression.

In summary, our analysis demonstrates that MeDeCom is a broadly applicable reference-free tool allowing us to explore complex data sets for confounding variables and, thus, to improve the biological interpretation of large-scale DNA methylation data sets. For the pilot demonstration, we exclusively used Infinium 450k data. In principle, MeDeCom is applicable to any complex methylome data set. However, since MeDeCom requires a low level of technical noise and a high level of biological variation, we suggest that the method is applied to carefully controlled data sets that fulfill such requirements. A high standard technical preprocessing of 450k array data minimizes possible pitfalls of quality, technical batch effects, or other non-biological issues. We, therefore, recommend using data after passing them through available bioinformatic pipelines (see, e.g., [55] or [56]).

## Methods

### MeDeCom element I: mixture model for DNA methylation measurements

Let *D*∈ [ 0,1]^*m*×*n*^ be the matrix of absolute methylation values of *m* CpGs obtained from *n* multicellular specimens, with *m* typically being much larger than *n*. Here, entry *D*_*ij*_ represents the methylation level for CpG *i* for specimen *j*, with *i*=1,…,*m* and *j*=1,…,*n*. We consider an approximate low-rank model for *D* assuming that the cell populations of samples consist of a finite number of sub-populations each contributing a distinctive methylation profile. We also assume that population mixtures are similar but slightly variable across biological samples collected in the same manner. Both assumptions suggest that the methylation profiles of the samples are a weighted average (mixture) of the methylation profiles associated with the underlying cell types, where the weights equal the proportions of these cell types. Note that we verified this concept in our analysis with artificial cell mixtures. Our matrix factorization model,

1 $$ D = TA + E,  $$

represents this concept where *T*∈ [ 0,1]^*m*×*k*^ represents the methylation profiles of *k* cell prototypes or other recurrent variables (in most cases representing a specific cell type) and $A \in \mathbb {R}_{+}^{k \times n}$ such that *A* ^⊤^ **1**_*k*_=**1**_*n*_ (i.e., the entries of *A* are non-negative and its columns sum to one). Entry *T*_*is*_ equals the methylation profile of CpG *i* of prototype *s*, with *i*=1,…,*m* and *s*=1,…,*k*, while *A*_*si*_ equals the relative abundance (proportion) of prototype *s* in specimen *i*. The matrix *E* represents errors, capturing model misspecification and noise arising from the measurement process. Note that the biologically motivated constraints for *T* and *A* distinguish our model from other low-rank models as they are used for adjustment of the phenotype association analysis [39–41]. Notably, (1) can be seen as an approximation of a more general constructive or exact model while the emerging approximation error can be estimated analytically (see Fig. 1 a and Additional file 5: Supplementary Note 1).

### MeDeCom element II: model fitting

Using a straightforward least-squares approach to fit model (1), yields the optimization problem:

2 $$ \begin{array}{ll} \min_{T,A} ||{D - TA}||_{F}^{2} = &\sum_{i=1}^{m} \sum_{j = 1}^{n} (D_{ij} - (TA)_{ij})^{2} \\ \text{subject to} \;\;& 0 \leq T_{is} \leq 1 \;\, \forall i,s \\ & A_{sj} \geq 0 \;\, \forall s,j \\ & \sum_{s = 1}^{k} A_{sj} = 1 \;\, \forall j. \end{array}  $$

Here and in the following, ∥.∥_*F*_ denotes the Frobenius norm of a matrix, defined as the square root of the sum of squares of its entries. We may think of the above problem as an instance of blind source separation, a task that has been well studied in signal processing [57]. The attribute blind expresses that the source signals, as represented by the columns of the matrix *T*, are unknown, as opposed to when they are given in advance and only the mixture coefficients in *A* need to be recovered.

The minimization problem in (2) is not jointly convex in *T* and *A*. As a result, one cannot hope to converge always to the global optimum; in fact, it has been shown that constrained matrix factorization problems of this form are computationally hard in general [58].

Once *T* or *A* is given, the problem (2) leads to a convex quadratic program. This property is the basis of *alternating minimization*, a common (heuristic) approach for fitting matrix factorization models where one alternates minimization w.r.t. *T* for fixed *A* and vice versa [59]. While lacking theoretical guarantees, alternating minimization often works very well in practice.

Note that independently of our work, Houseman et al. [42] recently proposed RefFreeCellMix, an approach like (2). A rather minor difference is that in RefFreeCellMix, the equality constraint, $ \sum _{s = 1}^{k} A_{sj} = 1$, is replaced with an inequality constraint, $\sum _{s = 1}^{k} A_{sj} \leq 1$. Thus, the components of *A* estimated by RefFreeCellMix cannot be interpreted as the proportions of the corresponding methylation profiles. Moreover, we will argue in the following that the direct use of approach (2) ignores valuable prior biological information about the problem, which leads to suboptimal solutions. This, in turn, has an adverse effect on the estimation of the proportions *A* and the methylation profiles *T*, leading to considerably worse solutions.

The main problem of (2) is ill-posedness. In general, there are multiple optimal solutions to (2) (excluding those generated by column and row permutations in *T*, respectively, *A*), as can easily be seen from geometric considerations (see Fig. 1 c). In geometric terms, problem (2) can be re-phrased as follows: find a set of *k* points {*t*_1_,…,*t*_*k*_}⊂ [ 0,1]^*m*^ corresponding to the columns of *T* such that their convex hull $\mathcal {T} = \{y \in \mathbb {R}^{m}: y = \sum _{s = 1}^{k} \lambda _{s} t_{s}, \; \lambda _{s} \geq 0 \; \forall s, \; \sum _{s=1}^{k} \lambda _{s} = 1\}$ minimizes the sum of squared Euclidean distances of the data points {*D*_:,1_,…,*D*_:,*n*_} to that convex hull. As shown in Fig. 1 c, one can easily construct problem instances for which it is possible to extend or shrink $\mathcal {T}$ while keeping the least-squares objective (essentially) unchanged. Note that a solution from RefFreeCellMix or one from our model without the regularizer (*λ*=0) will be far away from the ground truth and, thus, have gross errors both in the estimation of the proportions *A* as well as the profiles *T*.

To deal with this ambiguity, we suggest complementing the least-squares objective with a biologically plausible regularizing term pushing the points {*t*_1_,…,*t*_*k*_} towards the vertex set of [ 0,1]^*m*^, i.e., the set of binary vectors {0,1}^*m*^. The rationale behind this is as follows. Recall that the columns of *T* take the role of methylation profiles of prototypes, which in typical cases represent a (near) homogeneous sub-population of cells. Depending on the homogeneity of the sub-population, the methylation profile of the corresponding prototype may be close to binary since at the level of a single cell, methylation profiles are exactly binary (methylated vs unmethylated) when ignoring the comparatively rare case of half-methylation. Incorporating this structure contributes significantly to the success in finding biologically meaningful matrices *T* and *A*. Specifically, we consider the following regularized least-squares criterion:

3 $${} \begin{aligned} &\min_{T,A} \|D - TA\|_{F}^{2} + \lambda \sum_{i = 1}^{m} \sum_{s=1}^{k} \omega(T_{is}), \text{with} \,\omega(x) = x(1 - x),\\ &\text{subject to~} 0 \leq T_{is} \leq 1 \;\, \forall i,s \\ &\qquad \qquad A_{sj} \geq 0 \;\, \forall s,j \\ & \qquad \qquad \sum_{s = 1}^{k} A_{sj} = 1 \;\, \forall j, \end{aligned}  $$

where *λ*≥0 is a hyperparameter. Note that *ω*:[ 0,1]→[ 0,1] is a quadratic function symmetric around its mode 0.5 (i.e., *ω*(*x*)=*ω*(1−*x*)) and vanishes at the boundary points 0 and 1. The additional regularization term in (3) acts as a soft binary constraint depending on the parameter *λ*. For *λ* sufficiently large, any minimizer $(\widehat {T}, \widehat {A})$ of (3) must satisfy $\widehat {T}_{is} \in \{0,1\}$ for all *i,s*. We stress that the proposed form of regularization is much better suited to the given problem than the popular lasso (*ℓ*_1_ regularization with *ω*(*x*)=|*x*|), which promotes zeroes but discourages ones, which has little meaning for the given problem from a biological perspective.

We would like to stress again that the introduction of this regularizer constitutes a key prerequisite for getting biologically meaningful solutions for matrices *T* and *A*. While (2) and RefFreeCellMix work reasonably well if the methylation profiles of the pure cell types are present as samples in the data matrix *D*, this approach can fail completely if the measured samples consist only of mixtures of cell types, as shown in the artificial NeuN ^+/−^ mixture experiment. The reason for the bad performance of RefFreeCellMix is that it basically interprets the mixtures (0.3,0.7) and (0.7,0.3) as columns of *T*, whereas the regularizer proposed in the present paper pushes *T* towards 0 (respectively 1), and, thus, can estimate the correct profiles and their proportions accurately.

From the computational standpoint, the extra term in (3) poses an additional challenge compared to (2), as the function *ω* is non-convex (in fact, it is concave). As a consequence, when using the alternatization scheme mentioned above, one has to bear in mind that optimizing *T* for fixed *A* is no longer a convex quadratic program, but a so-called difference of convex program in virtue of the concavity of *ω*. The concave–convex procedure [60, 61] can be employed to generate a sequence of iterates ensuring the monotonic descent of the objective function before reaching a stationary point. As detailed in Algorithm 1, it is straightforward to integrate this approach into the alternating optimization scheme.



The main computational efforts go into the successive solution of the convex quadratic optimization problems **optT** and **optA**, which can be done by a variety of efficient solvers. Updating *T* follows the concave–convex procedure in which the concave part of the objective (here given by *h*(*T*)) is repeatedly linearized, yielding a sequence of convex surrogate minimization problems.

### MeDeCom element III: parameter selection

The mixture model (1) and the fitting algorithm (Algorithm 1) involve two free parameters to be provided by the user. The inner dimension *k* of the matrix product *TA*, *k*≤ min{*m,n*} in (1), equals the number of DNA methylation prototypes used to model the given data. The regularization parameter *λ* determines how strongly the entries of $\widehat {T}$ are encouraged to take values in {0,1}. The choice of *k* can be guided by prior (biological) knowledge about the possible composition of the underlying mixture. However, to select the optimal values of *k* and *λ*, we developed a cross-validation procedure.

#### Cross-validation

Typical approaches to cross-validation in matrix factorization are (a) leaving out columns, (b) leaving out rows, and (c) leaving out both rows and columns [62]. We decided to use (a) since it leads to a straightforward scheme as displayed in Algorithm 2. For each fold, a subset of the samples is left out. Thereby, the column-reduced data matrix *D* ^in^ is factorized as if one were given the full matrix. The resulting left factor $\widehat {T}^{\text {in}}$ is used to fit the left-out columns in *D* ^out^ as $D^{\text {out}} \approx \widehat {T}^{\text {in}} \widehat {A}^{\text {out}}$. The squared error of that approximation or CVE is saved and finally combined with the errors from other folds.



#### Selecting *k*

The choice of *k* is critical for the good performance of our model. In some instances, such as for the synthetic mixtures, the number of cell populations are known and the optimal selection of *k* is straightforward. However, for most biological samples, prior knowledge of cell-type composition and other variables is not available or can only be estimated. Moreover, a number of other variable effects, such as age, gender, genetic background, allelic variations etc., have to be included to obtain an interpretable LMC separation. We observe that *k* should be chosen such that the estimation error and the approximation error in model (1) are roughly balanced. The former results from noise and is incurred when fitting the model to the data, while the latter is a consequence of model misspecification, which, as discussed above, is inevitable for limited *k* given the many possible sources and levels of variance.

Statistically the selection of *k* is related to the selection of numbers of components in a principal component analysis (PCA). In fact, the matrix factorization model (1) can be seen as a method of linear dimension reduction applied to *D*. A common computational approach to PCA is singular value decomposition (SVD), which yields a matrix factorization of rank *k* of *D* by discarding all singular vectors not corresponding to the top *k* singular values. A notable advantage of our scalable model (1) over the truncated SVD/PCA is its direct interpretability at a biological level, which is achieved by putting suitable constraints on the two factors *T* and *A*.

For a fixed value of the parameter *λ*, the data-fitting term of the factorization problem (3) decreases as *k* increases. The approximation error of the factorization model decreases since with more columns in *T*, one has a better chance of capturing differences between the cluster methylomes. At the same time, the estimation error increases as the additional degrees of freedom favor over-adaptation to noise. A suitable choice of *k* balances both effects. The use of cross-validation is intended to achieve this balance by tracing the CVE over a grid of values for *k* and selecting the one corresponding to the minimum. The final choice of *k* is made by combining visual inspection of the cross-validation results and available prior information about the most likely number of underlying methylation signatures.

#### Selecting *λ*

In our example in Fig. 1 b, the regularization parameter *λ*, which balances the trade-off between the data fidelity term and the data-independent regularization term, has a crucial influence on the solution of the factorization problem (1) delivered by Algorithm 1. Since there is, in general, no objective criterion to assess the suitability of each solution at a biological level, we use CVE, as for the parameter *k*. Determining a minimum CVE for *λ* is difficult as that parameter takes values in a continuous domain, namely the non-negative real line. To approach this, we perform a two-stage grid search, starting with a coarse grid and then concentrating on a smaller range covered by a finer grid. Details of the procedure are outlined in Algorithm 3. At the beginning of each of the two rounds of the grid search, Algorithm 3 is run for each grid point of *λ* using multiple (≈50) random initializations. As the solutions corresponding to nearby grid points can be expected to be similar, we complement random initializations with a smoothing scheme in which the solutions of the five preceding and the five subsequent grid points are used for initialization.



#### Computational performance

When *m*≫*n*, the computational burden is dominated by the optimization step over *T*, which scales in the worst case as *O*(*n m k* ^3^), where *O*(*k* ^3^) is the worst-case performance in solving a quadratic program of size *k*, which in practice often behaves better. However, the optimization of rows of *T* can be done independently and, thus, we have parallelized this step, leading to an almost linear speed-up on multi-core machines. Moreover, we have parallelized all the independent runs done for cross-validation and used to find a good regularization parameter. While still computationally demanding, the method is in this way scalable to large data sets, both for the number of CpG sites *m* and number of samples *n*. RefFreeCellMix is faster than MeDeCom as it does not have to test for different regularization parameters. However, a single factorization for *λ*=0 is faster in MeDeCom.

### LMC matching

As a first interpretation level, we propose matching MeDeCom LMC results of unknown samples to reference profiles, which can be either methylomes of purified cell types or other LMCs. Given a matrix of *k* LMCs $\widehat {T}$ estimated from a data set *D* and a matrix of *k* ^⋆^ reference profiles *T* ^⋆^, we first selected a set of rows $\mathcal {R}$ corresponding to the overlap of CpGs present in both $\widehat {T}$ and *T* ^⋆^. We then computed the matrix *S*=(*S*_*i,j*_) of Pearson correlation coefficients between all pairs of vectors $\widehat {T}_{\mathcal {R},i}$ and $T^{\star }_{\mathcal {R},j}$. We consider LMC $\bar {i}$ as a match to reference profile $\bar {j}$ if $S_{\bar {i}, \bar {j}}=\max _{i} {S_{i,\bar {j}}}$. We considered the matching unambiguous when $S_{\bar {i}, \bar {j}}=\max _{j} {S_{\bar {i},j}} = \max _{i} S_{i,\bar {j}}$ for all such matching pairs $(\bar {i}, \bar {j})$. In most of the cases, we observe better matching when both $\hat {T}$ and *T* ^⋆^ are centered, i.e., $(1/k) \hat {T}\mathbf {1}_{k}$ (respectively $(1/k^{\star }) T^{\star } \mathbf {1}_{k^{\star }}$) is subtracted from each column. To compare sets of prototypes corresponding to different parameter settings, we normalize the total number of unambiguously matching prototypes by the achievable maximum, which yields a score *ε*∈[0,1] given by $\epsilon = 1/\min (k,k^{\star }) \ |\big \{ (\bar {i}, \bar {j}) \in \{1,\ldots, k \} \times \{1,\ldots,k^{\star }\} : S_{\bar {i}, \bar {j}}=\max _{j} S_{\bar {i},j}\ \text {and}\ S_{\bar {i}, \bar {j}} = \max _{i} S_{i,\bar {j}} \big \}|$.

On the next alternative level, we propose a combined clustering analysis of LMC prototypes and reference profiles. For that, we composed a matrix $T^{\dagger } = [\widehat {T}_{\mathcal {R},:} \; T^{\star }_{\mathcal {R},:}]$. We also computed a correlation matrix *S* ^*†*^ analogously to *S*, and used it as a similarity matrix for agglomerative hierarchical clustering with average linkage (procedure hclust in the R package *clust*).

### Functional annotation of LMC-specific CpG positions

On a third level, we propose a functional annotation of the recovered LMCs by selecting component-specific CpG positions using a fixed methylation difference threshold *θ*. We consider a CpG position *l*∈{1,…,*m*} to be specific to component *i* if $|\widehat {T}_{l,j}-\sum _{j \ne i} \widehat {T}_{l,j}| > \theta $. We investigate each set $\mathcal {L}_{j}$ of all such CpGs with respect to enrichment of annotation categories using GREAT [51]. In general, we use the default definition for a functional domain of a gene, with a maximal distance of 10 kb upstream or downstream of the transcriptional start site (the “two closest genes” option in GREAT).

### Reference-based estimation of cell-type proportions

If a matrix *T* of *k* prototype methylomes is available, e.g., experimentally obtained using cell separation methods, one can estimate a corresponding matrix of mixture proportions by solving sub-problem **optA** in Algorithm 1. From here onwards, we refer to this method as regression, and we apply it for reference-based estimation of mixture proportions whenever the reference methylomes are available. This form of proportion estimation is like a method called constrained projection proposed for the same purpose in [33]. The important difference is, however, that the analogue of the matrix *T* in that method is constructed from the selection of a comparatively small set of cell-type-specific marker CpGs. In the following, we compare to its proportion estimates whenever appropriate.

### Application of RefFreeCellMix

We performed reference-free deconvolution with the method RefFreeCellMix by Houseman et al. [42] using the R package RefFreeEWAS. In accordance with the original publication of the method [42], we applied it to the 20,000 most variable CpG positions from the methylation matrix, unless the total number of rows was less, in which case we used the full matrix. In the former case, we used the available option to obtain the estimates of the methylation components for all CpGs as the final step of the deconvolution procedure (supplying the complete data matrix as argument Yfinal).

### Simulations

For the performance analysis, we generated simulated DNA methylation data by mixing measured profiles of isolated cell types in controlled proportions and adding varying levels of Gaussian noise. An *m*×*n* matrix of DNA methylation values *D*_sim_ was generated according to the model in (1).

The underlying matrix of LMCs $\phantom {\dot {i}\!}T \in [0,1]^{m \times k_{\text {sim}}}$ was obtained by averaging methylation profiles for *k*_sim_ purified blood cell types from six donors in the Reinius et al. study [44]. We tested four different constellations of blood cell types:

- *k*_sim_=2 with two distant cell types (neutrophils and CD4+ T cells).
- *k*_sim_=2 with two similar cell types (neutrophils and monocytes).
- *k*_sim_=3 with two similar cell types and one distant from the first two (neutrophils, monocytes and CD4+ T cells).
- *k*_sim_=5 with all major blood cell types, excluding eosinophils and B cells.

The columns of the matrix of mixture proportions *A* were sampled from a Dirichlet distribution commonly used to model distributions over the probability simplex. The distribution had *k*_sim_ parameters $v\alpha _{1},\dots,v\alpha _{k_{\text {sim}}}$. The simplex base $\alpha _{1},\dots,\alpha _{k_{\text {sim}}}$, $\sum _{i} \alpha _{i}=1$, was chosen to model the prior expectation for the mixing proportions in a typical individual. We tested two scenarios: on average equal (uniform) proportions across individuals, i.e., *α*_*i*_=1/*k*_sim_, *i*=1,…,*k*_sim_, and a setting where some concentration parameter values were much larger than others, which comes closer to the situation one encounters for whole blood. The scaling factor *v* was used to control the variability of the mixing proportions, with *v*=1 yielding highly variable, *v*=10 moderately variable, and *v*=100 marginally variable proportions across individuals. Finally, the additive noise term *E* was generated by sampling *mn* values from a Gaussian distribution with mean 0 and standard deviation 0.05, 0.1, and 0.2 to simulate low, moderate, and high levels of noise, respectively.

To simulate true methylation effects of average size *δ* for *m*_*e*_≪*m* CpGs in cell type *l* under a simple case vs control setting, the source cell-type-specific methylation profiles of the affected samples (cases) were changed to mimic DNA methylation differences. More specifically, a set $\mathcal {C}_{e}$ of affected CpGs randomly sampled from 1,…,*m*, and a matrix *T* ^*e*^ was obtained so that $T^{e}_{l,u} = T^{e}_{l,u} + \mathcal {N}(\delta,\sigma) \mathcal {I}_{\mathcal {N}(0,1)>0}$ where $u \in \mathcal {C}_{e}$. The simulated effect for the proportion of cell type *l* was introduced by changing parameter *α*_*l*_ of the Dirichlet distribution for one sample group only.

### Infinium 450k data

#### Public Infinium 450k data sets

The publicly available data sets used to validate the factorization approach are summarized in Table 1. To test MeDeCom for blood-based data, we used one reference data set and data from two large whole-blood-based studies. The data set from Reinius et al. contains profiles of purified blood cell types, as well as mixed samples with known cell counts [44]. In addition, we used data from a large rheumatoid arthritis EWAS with 354 cases and 337 controls [35]. Finally, we validated the whole-blood results in the data from the EPIC Italy prospective cohort, which provided 845 Infinium 450k measurements [45]. Neuronal data sets were obtained from one reference study and one large AD cohort. As a reference, we used data from the CETS study [37], which contained in total 145 Infinium 450k profiles of various neuronal samples from major depression disorder patients and healthy controls, such as cortical NeuN ^+^- and NeuN ^−^-enriched cell populations, nine artificial NeuN ^+/−^ titration mixtures, as well as 20 intact frontal cortex samples. For validation, we used data from a recent AD study [19].

#### Processing and preparation of the Infinium 450k data

The raw Infinium 450k data were collected as IDAT files or, if the latter were not available, from probe-wise intensity matrices (Illumina Genome Studio reports). Loading and primary processing, such as intensity summarization and methylation ratio (*β* value) calling, was performed with the RnBeads package [55]. We used *dasen* as the primary normalization method [63]. We used several layers of filtering criteria to eliminate low-quality probes. We required each methylation call to be supported by at least five Infinium beads. Since too low and too high probe intensity may indicate measurement problems, we discarded CpGs where the raw intensity for either methylated or unmethylated probes was below 0.1 or above 0.9 quantiles of the total intensity distribution in the respective channel. To diminish the effects of genetic variation, we also discarded CpGs with probes that overlapped with annotated single-nucleotide polymorphism positions (dbSNP132 entries with MAF >0.05, as defined in the RnBeads.hg19 annotation) along the whole probe sequence.

#### Adjustment of the phenotype association analysis

For consistency with the published results, we performed the association analysis using the code that we obtained from the authors of the ReFACTor paper [41]. For the unadjusted analysis, a logistic linear model was fitted for each CpG site, with the phenotype (rheumatoid arthritis status) as a response variable and methylation level as the only predictor. The *T* test of the predictor variable coefficient being different from zero was used as the test of the association. For the adjusted analysis, first, the ordinary linear model was fitted to the methylation data for each CpG using the common covariates, such as age, gender, smoking status, and the experimental bath, as predictors. The residuals of this model were then used to fit the phenotype model instead of the actual methylation values. The adjustment for cell composition was performed either via a specialized statistical procedure (RefFreeEWAS [39] and Fast-LMM-EWASher [40]) or by including additional covariate variables reflecting the compositional variation. In the reference-based adjustment, unconstrained cell-type contribution estimates obtained with the Houseman et al. method [33] were added to the covariate list. For RefFreeEWAS and Fast-LMM-EWASher, no custom modeling was performed, but the data and common covariates were supplied directly to the published implementations and the output *P* values were used for the comparison. When adjusting using ReFACTor and RefFreeCellMix, columns of the recovered matrices *R* and *Ω* were included, respectively. For MeDeCom, the LMC proportions were used as covariates. To decrease the complexity for large *k*, we considered including only *k* ^′^ LMCs with, on average, the largest proportions across all samples. The efficiency of the adjustment was assessed by comparing the observed distribution of *P* values to the expected one under the assumption that none of the tested null hypotheses are false (which corresponds to a uniform distribution).

## Additional files

Additional file 1 Supplementary Tables. PDF document with supplementary tables. (PDF 120 kb)

Additional file 2 Supplementary Figures. PDF document with supplementary figures. (PDF 2088 kb)

Additional file 3 CpGs used for the analysis of memory and naive B cells. A comma-separated value table file. (CSV 35 kb)

Additional file 4 LMC-specific CpG positions of the FC1 data set. A comma-separated value table file. (CSV 256 kb)

Additional file 5 Supplementary Text. PDF document with supplementary notes. (PDF 158 kb)

## Abbreviations

AD: Alzheimer’s disease
CVE: Cross-validation error
EWAS: Epigenome-wide association study
FACS: Fluorescence activated cell sorting
GEO: Gene Expression Omnibus
LMC: Latent (DNA) methylation component
MAE: Mean absolute error
MDD: Major depression disorder
NMF: Non-negative matrix factorization
PCA: Principal component analysis
RMSE: Root-mean-square error
SVA: Surrogate variable analysis
SVD: Singular value decomposition
MACS: magnetic activated cell sorting
WGBS: whole-genome bisulfite sequencing
DMR: differentially methylated region
MAF: minor allele frequency
