# Supplementary material for: MeDeCom: discovery and quantification of latent components of heterogeneous methylomes
Source: Genome Biol. 2017 Mar 24;18:55. doi: 10.1186/s13059-017-1182-6 (PMC5366155; doi:10.1186/s13059-017-1182-6)
Supplement: Supplementary file 1 — Supplementary Tables. PDF document with supplementary tables. (PDF 120 kb) [file 13059_2017_1182_MOESM1_ESM.pdf]

Table S1: Parameters for the simulation runs

		Methylation component schemes		
**Name**	2CompDistant	2CompSimilar	3comp1Distant2Similar	5Comp
k_{sim}	2	2	3	5
**Cell types**	Neutrophils<br>CD4+ T-cells	Neutrophils<br>Monocytes	Neutrophils<br>CD4+ T-cells<br>Monocytes	Neutrophils<br>CD4+ T-cells<br>Monocytes<br>CD8+ T-cells<br>NK-cells

	Proportion model ($k_{sim} = 5$)	
**Name**	"Uniform"	"Biological"
$\alpha_{Neutrophils}$	0.20	0.62
$\alpha_{Monocytes}$	0.20	0.23
$\alpha_{CD4+T-cells}$	0.20	0.05
$\alpha_{CD8+T-cells}$	0.20	0.07
$\alpha_{NK+T-cells}$	0.20	0.03

		Proportion variability	
**Name**	Low	Moderate	High
v	1	10	100
		Noise levels	
**Name**	Low	Medium	High
$\sigma_{\mathbf{noise}}$	0.05	0.1	0.2

Table S2: NeuN+ and NeuN- proportions in the ArtMixN data set

Sample	Propotion	
	NeuN-	NeuN+
121_Mix1(P2E1)_7786915074_R01C01	0.90	0.10
122_Mix2(P2E2)_7786915074_R02C01	0.80	0.20
123_Mix3(P2E3)_7786915074_R03C01	0.70	0.30
124_Mix4(P2E4)_7786915074_R04C01	0.60	0.40
125_Mix5(P2E5)_7786915074_R05C01	0.50	0.50
126_Mix6(P2E6)_7786915074_R06C01	0.40	0.60
127_Mix7(P2E7)_7786915074_R01C02	0.30	0.70
128_Mix8(P2E8)_7786915074_R02C02	0.20	0.80
129_Mix9(P2E9)_7786915074_R03C02	0.10	0.90

Table S3: FC1 data set ($k = 3$, $\lambda = 3 \cdot 10^{-3}$): overlap of the LMC1-specific genes with Moe *et al.* hypo-DMRs in coinciding with telencephalon development markers from Vicel *et al.*

TF	# Mo et al. CTs	LMC1 status
Ascl1	3	-
Dbx1	3	-
Ebf1	3	Hypermethylated
Ebf3	3	Hypermethylated
Egr3	3	-
Emx1	3	-
Emx2	3	-
Esrrg	3	-
Fezf2	3	Hypomethylated
Foxp2	3	-
Foxp4	3	-
Gbx2	3	Hypermethylated
Gli3	3	Hypermethylated
Gsx1	3	-
Gsx2	3	-
Hes1	3	-
Hes5	3	-
Id2	3	-
Id4	3	-
Isl1	3	-
Lef1	3	-
Lhx2	3	-
Lhx5	3	-
Lhx8	3	-
Lhx9	3	-
Mafb	3	-
Meis1	3	Hypomethylated
Meis2	3	-
Neurod1	3	-
Neurog1	3	Hypomethylated
Neurog2	3	-
Nkx6-2	3	-
Nr2e1	3	Hypomethylated
Nr2f1	3	Hypomethylated
Nr2f2	3	-
Olig1	3	-
Olig2	3	Hypomethylated
Otx1	3	Hypomethylated
Pax6	3	Hypomethylated
Pou3f2	3	Hypomethylated
Pou3f3	3	Hypermethylated
Rara	3	-
Sall3	3	-
Six3	3	-
Sox1	3	Hypomethylated
Sox4	3	-
Sp8	3	Hypomethylated
Sp9	3	-
Tle1	3	-
Tle3	3	-
Tle4	3	-
Tshz1	3	-
Vax1	3	-
Zfhx4	3	-
Zfp521	3	-
Zic1	3	Hypomethylated
Bcl11b	2	-
Dlx1	2	Hypomethylated
Dlx2	2	-
Dlx5	2	-
Dlx6	2	-
Eomes	2	-
Fezf1	2	-
Foxg1	2	-
Hmx3	2	-
Lhx6	2	-
Otx2	2	-
Prox1	2	-
Sox11	2	-
Tbr1	2	-
Zic5	2	Hypomethylated
Bcl11a	1	Hypermethylated
Nkx2-1	1	-
Pbx1	1	-
Zfp503	1	-
