# Supplementary material for: MeDeCom: discovery and quantification of latent components of heterogeneous methylomes
Source: Genome Biol. 2017 Mar 24;18:55. doi: 10.1186/s13059-017-1182-6 (PMC5366155; doi:10.1186/s13059-017-1182-6)
Supplement: Supplementary file 2 — Supplementary Figures. PDF document with supplementary figures. (PDF 2088 kb) [file 13059_2017_1182_MOESM2_ESM.pdf]

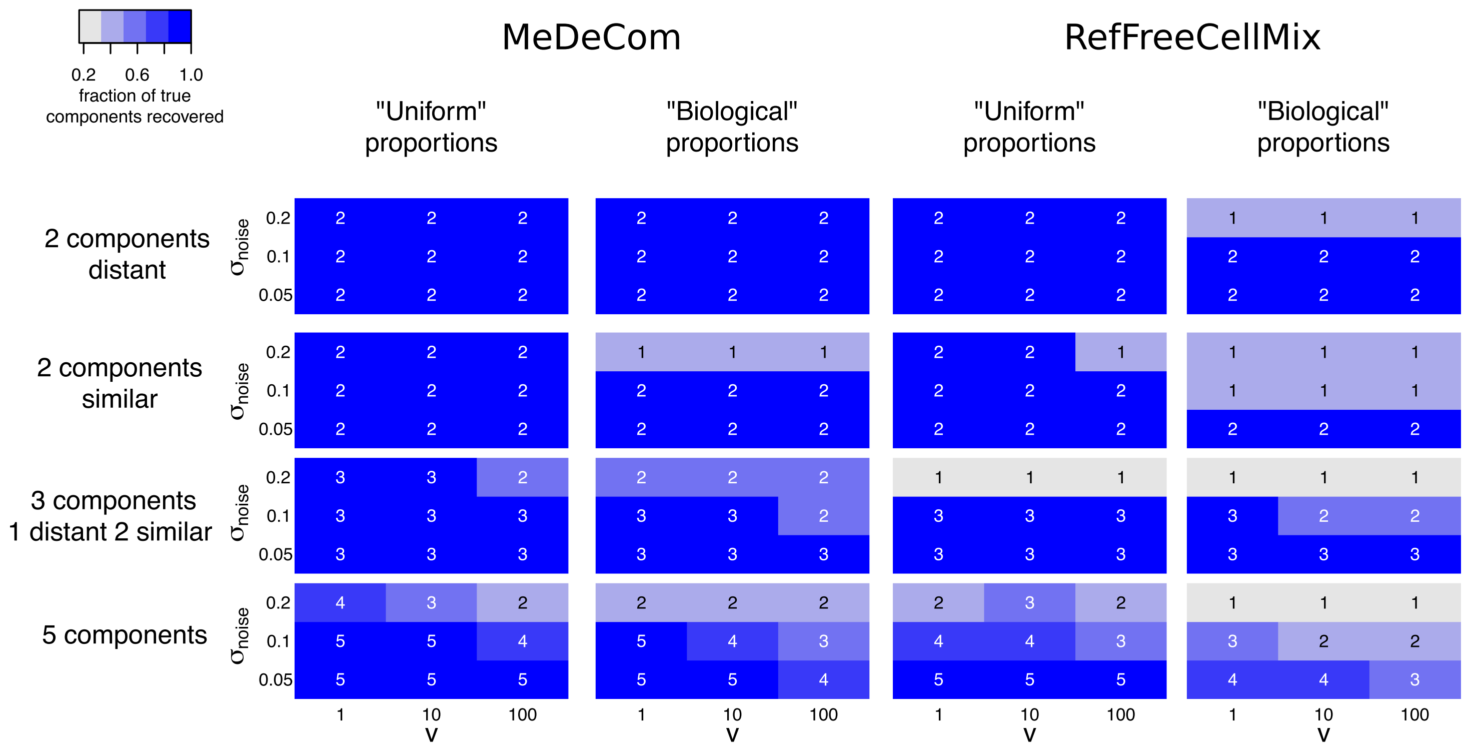

Figure S1: Efficiency of the component recovery in all simulated data sets. v is the scaling parameter used in proportion sampling (higher values of v correspond to lower variability of the mixture proportions), while σ_{noise} is the standard deviation of the additive Gaussian noise. The values show the maximum number of mutually matching $\hat{T}$ and $T^{\star}$ columns in the simulation analyses achieved for any of the tested k and λ values. Only the matches with Pearson correlation above 0.9 were considered. The color code shows the efficiency metric on $[0, 1]$, obtained by dividing the number of mutual matches by k_{sim}.

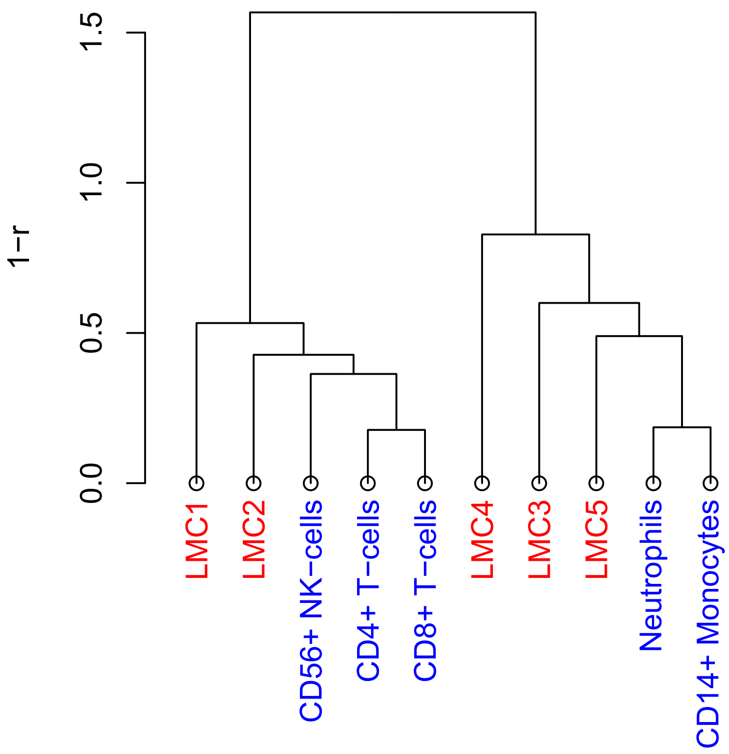

Figure S2: LMC recovery in a hard simulated test case with $k_{\text{sim}} = 5$, "biological" proportions with low variability ($v = 100$) and medium noise ($\sigma_{\text{noise}} = 0.1$), $k = 5$, $\lambda = 0.01$.

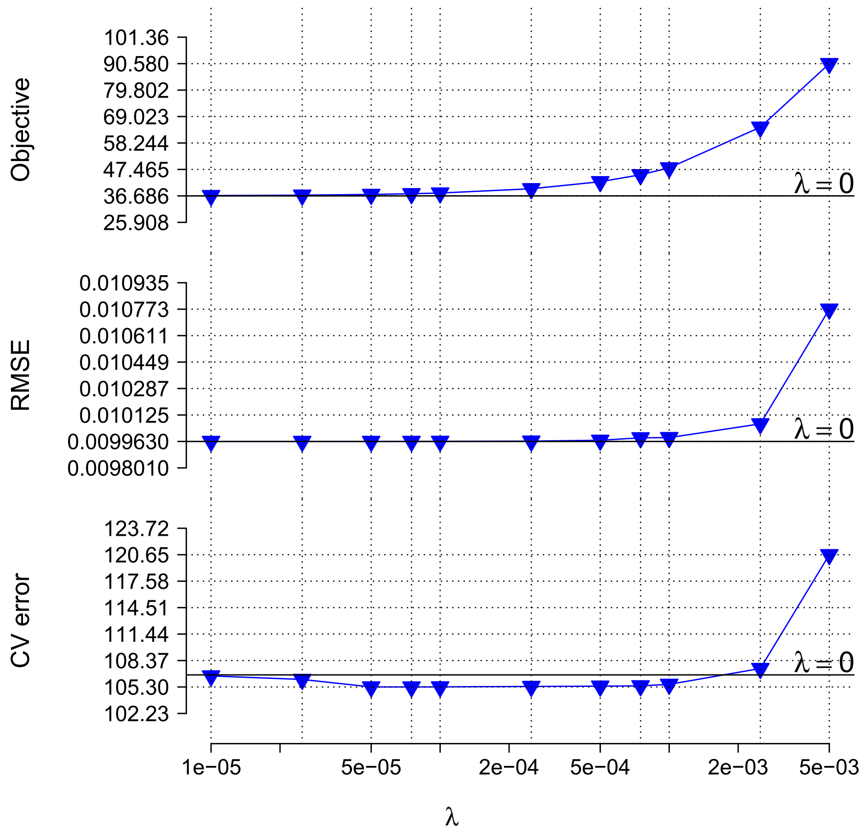

Figure S3: λ selection for 5 hardest mixtures of the ArtMixN data set ($k = 2$)

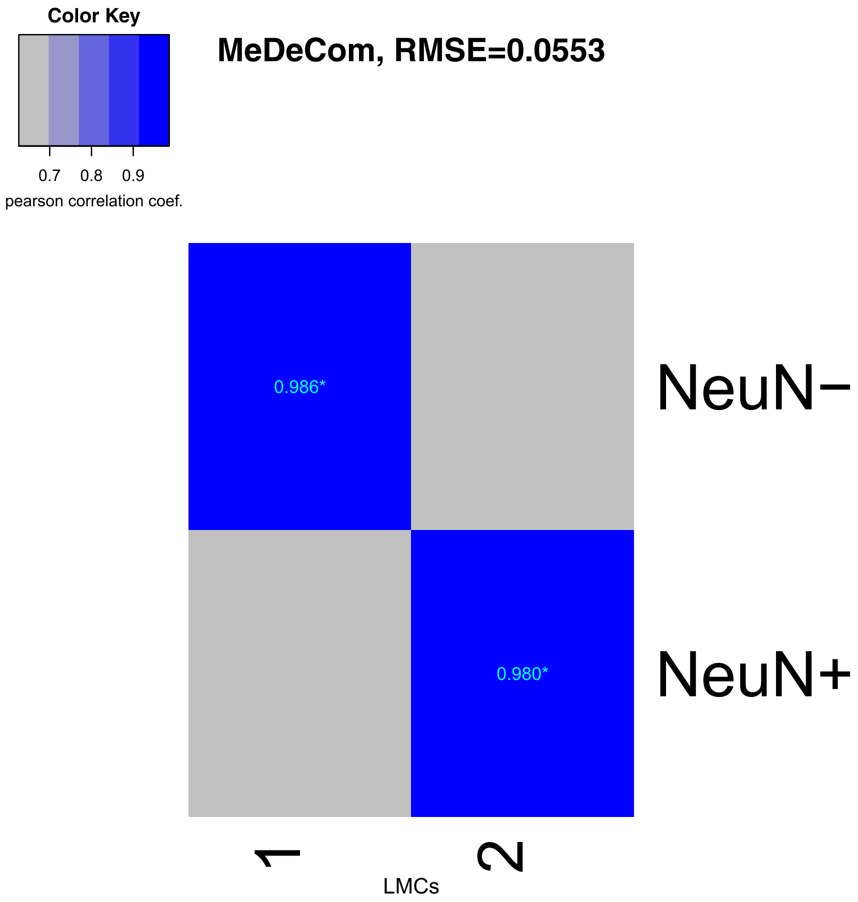

Figure S4: LMC recovery, ArtMixN data set, 5 hardest mixtures. 20,000 CpGs with the largest across-sample standard deviation are shown for better contrast.

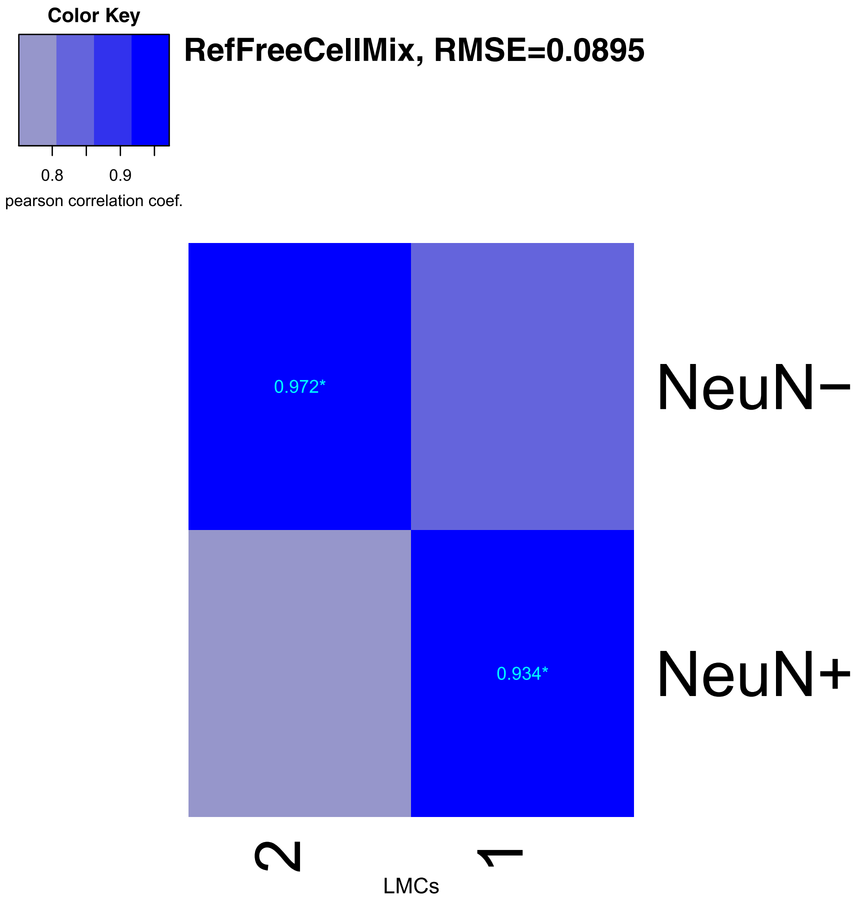

Figure S5: Results for the same setting as in Figure S4 obtained using RefFreeCellMix (Houseman *et al.*, 2016).

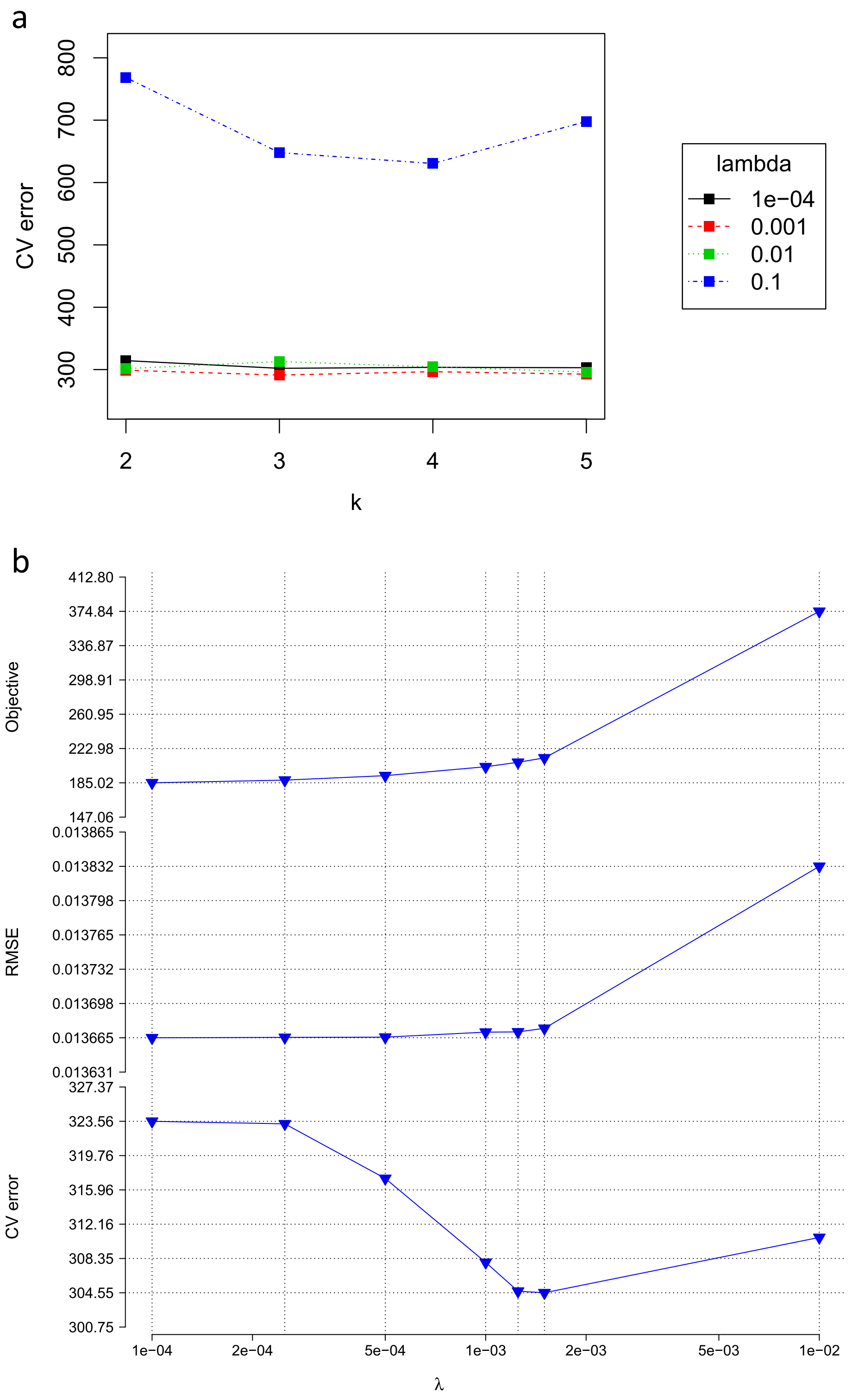

Figure S6: Parameter selection for the full ArtMixN data set ($k = 2$). a. k selection. b. λ selection.

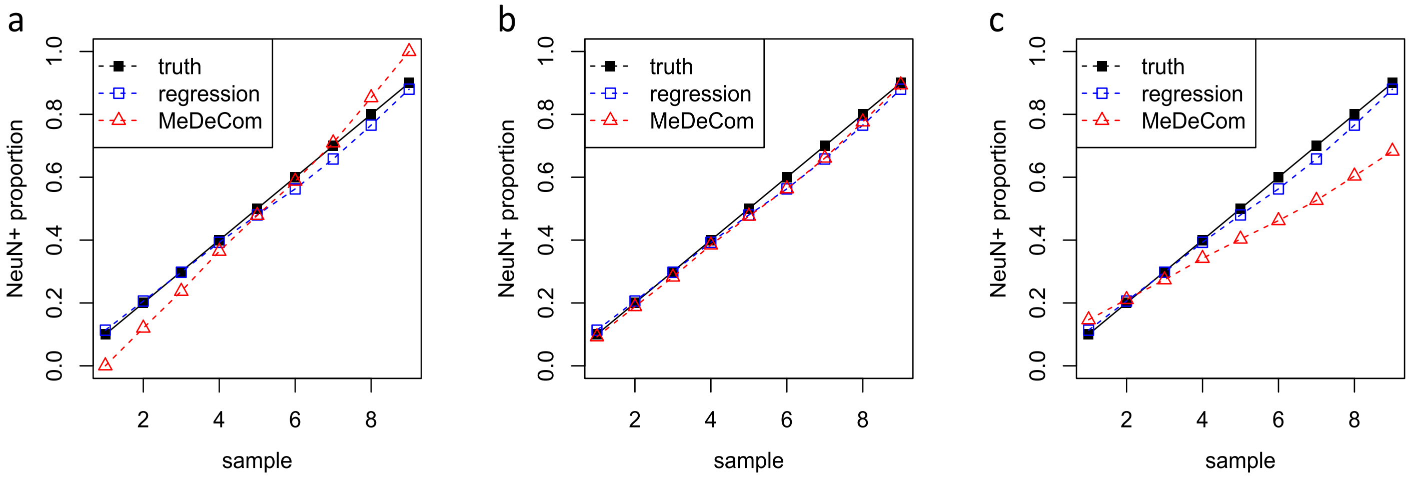

Figure S7: Regularization effects upon proportion recovery in ArtMixN data. a. $\lambda = 10^{-4}$. b. $\lambda = 10^{-3}$. c. $\lambda = 10^{-2}$.

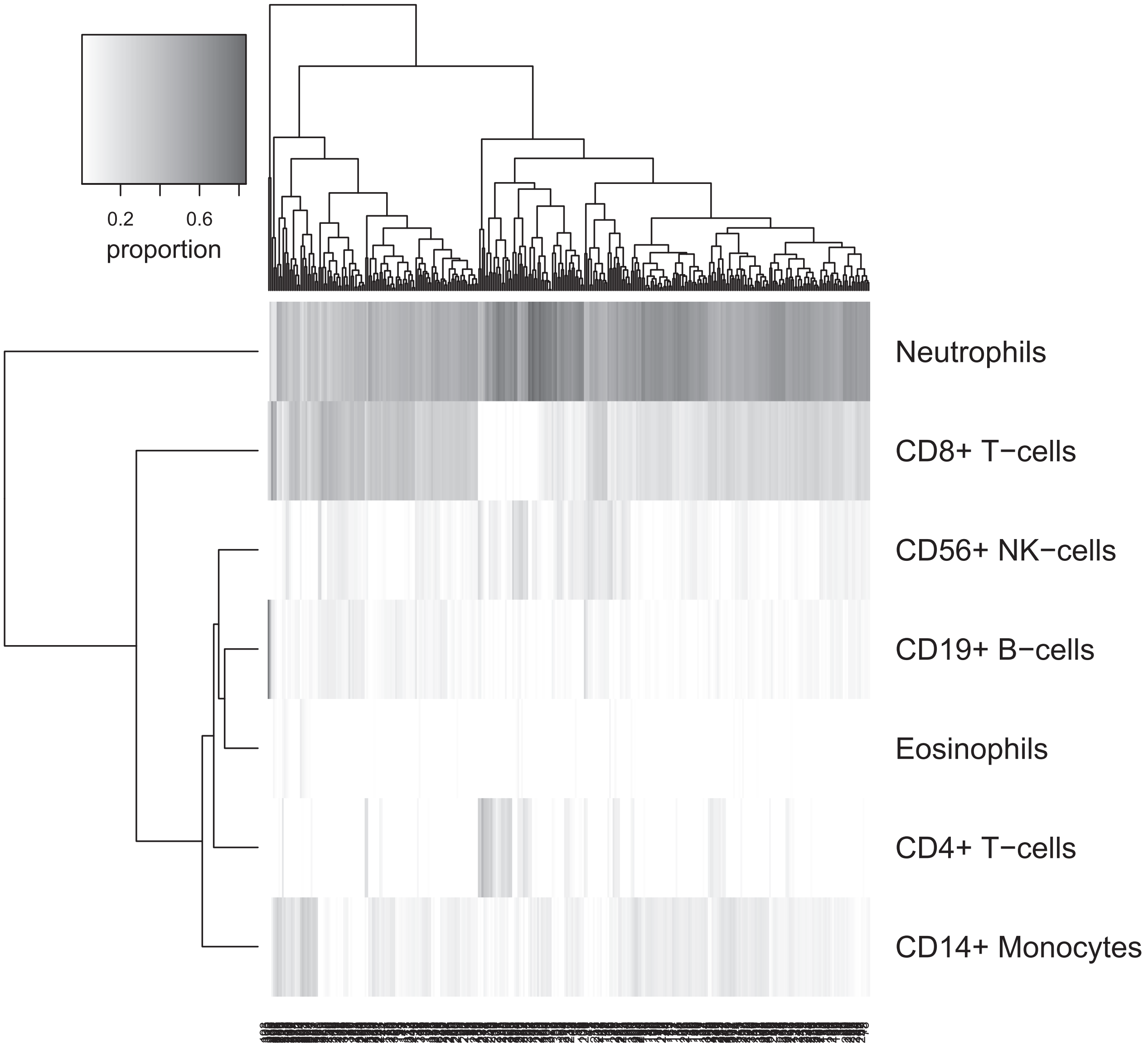

Figure S8: Regression estimated proportions of reference cell types in the control samples of the complete Liu *et al.* data set.

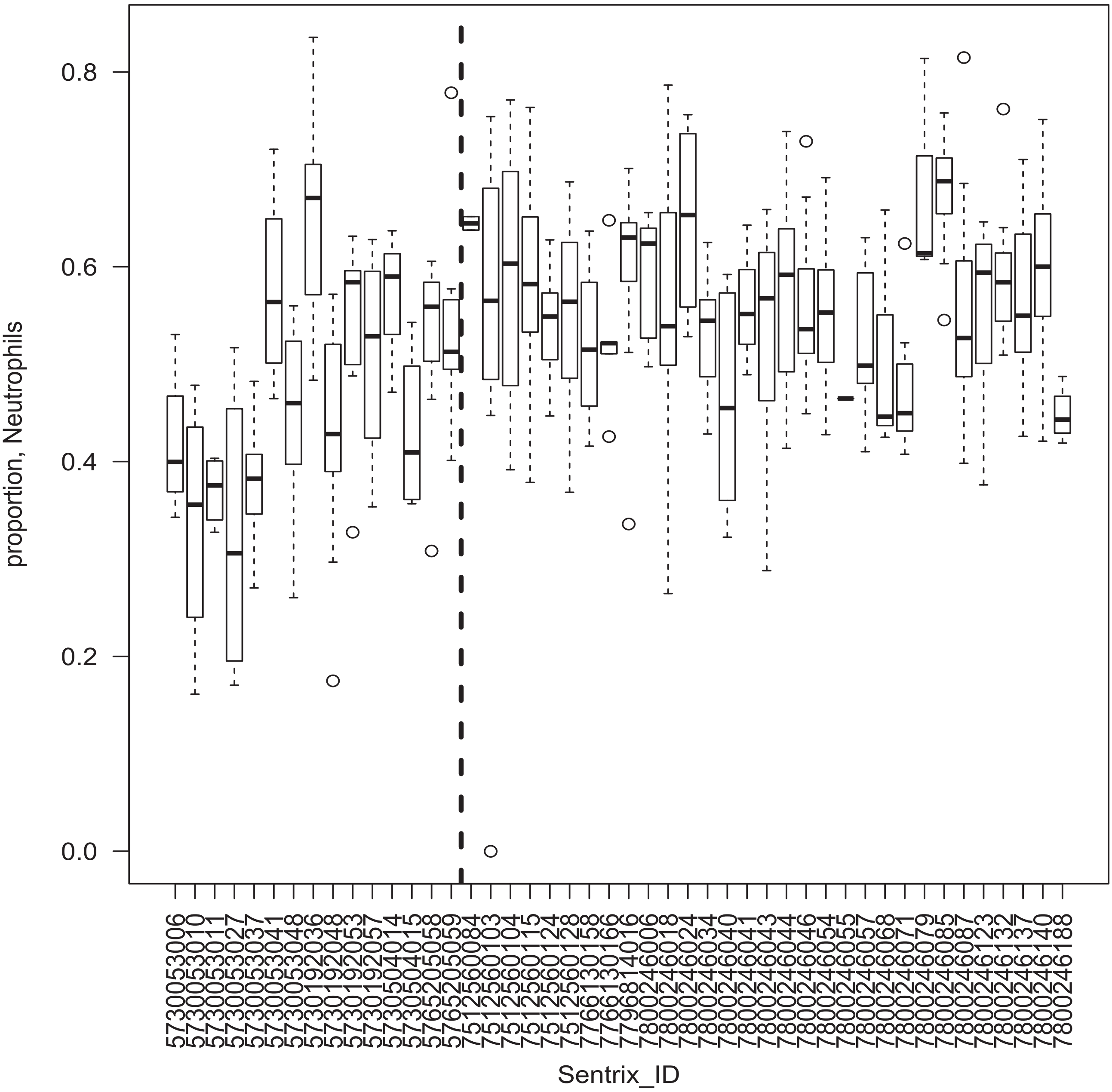

Figure S9: Estimated Neutrophil proportions in the complete Liu *et al.* data set, stratified by the 450k microarray plate (Sentrix_ID). One can easily notice a strong batch effect. The subsequent analysis was performed on a smaller technical batch of 87 samples with Sentrix_ID< 7512560084 (to the left from the dashed vertical line) comprising the WB1 data set.

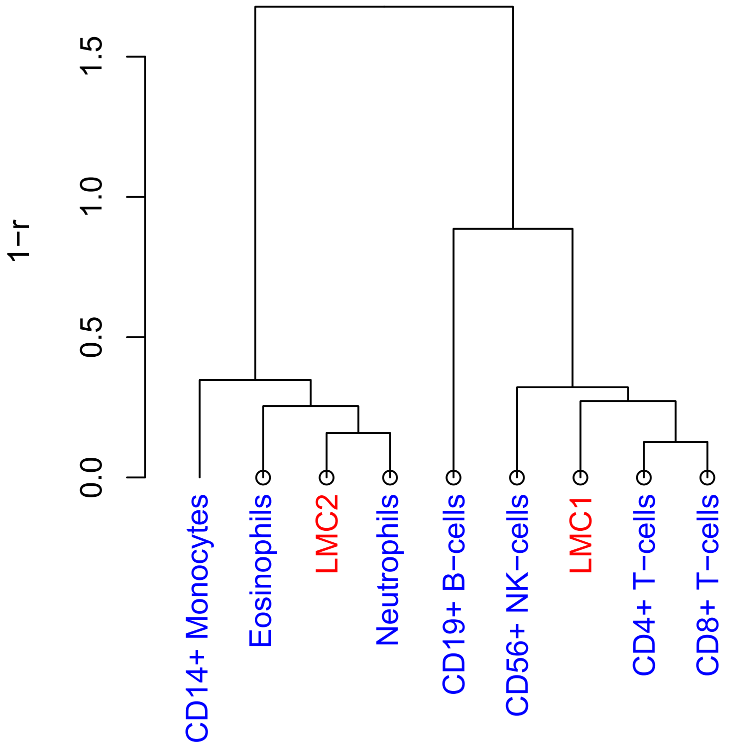

Figure S10: WB1 data set, matching of LMCs recovered with $k = 2$ and $\lambda = 0.01$

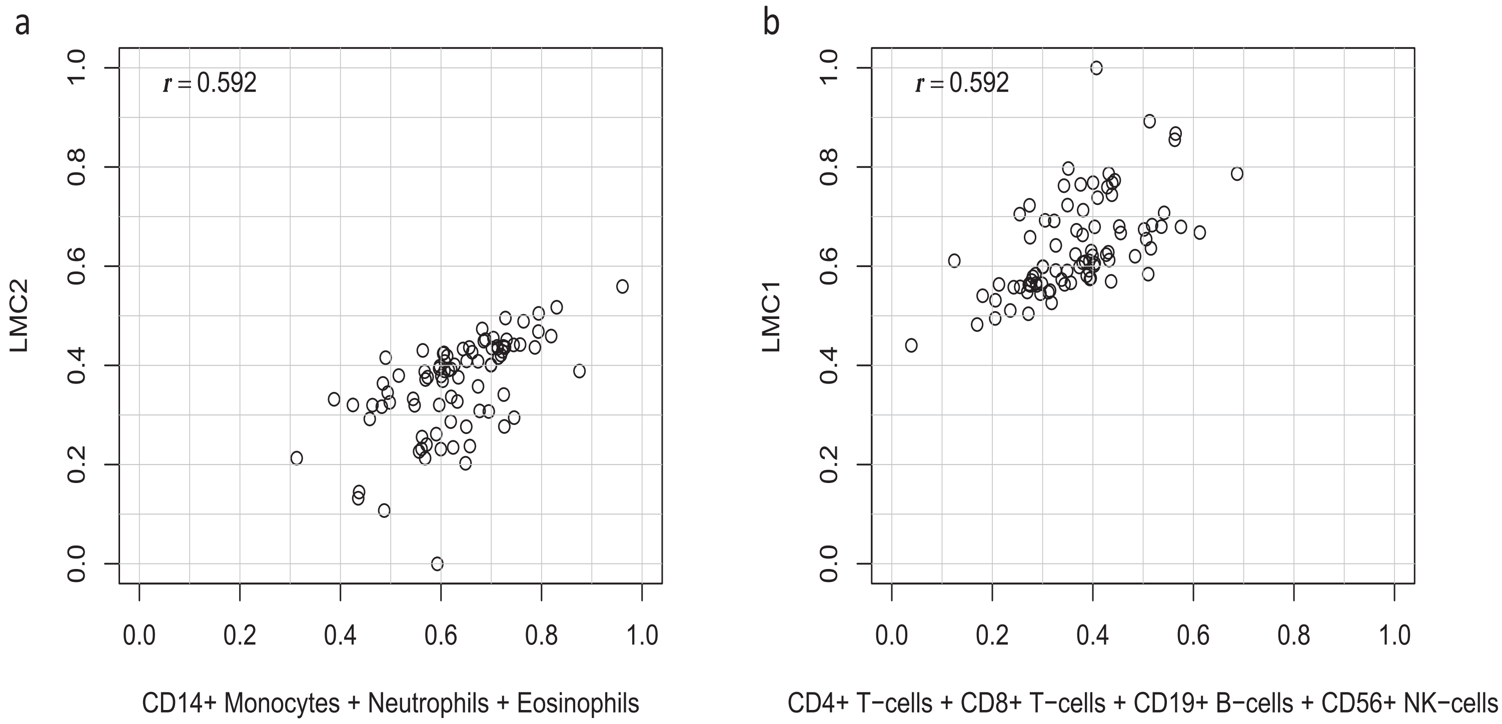

Figure S11: WB1 data set, comparison of the reference-based proportions of myeloid and lymphoid cell types and LMC proportions for the case with $k = 2$ and $\lambda = 0.01$. a. Myeloid cell types, including Neutrophils, Eosinophils and Monocytes. b. Lymphoid cell types, including T-cells, B-cells and NK-cells.

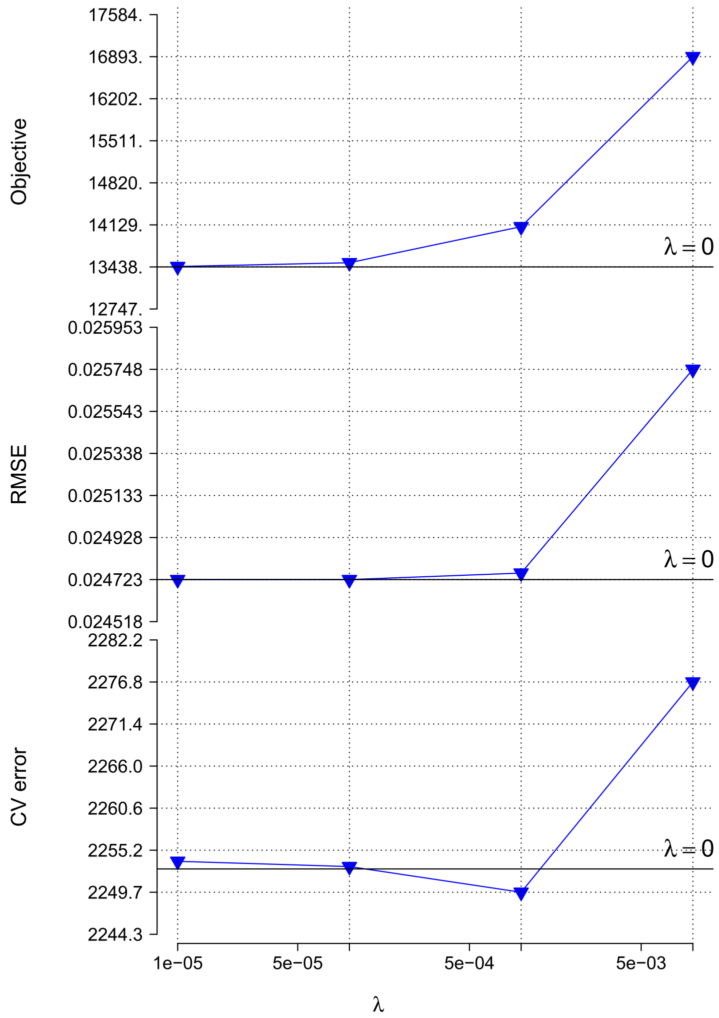

Figure S12: WB1 data set, λ selection ($k = 20$).

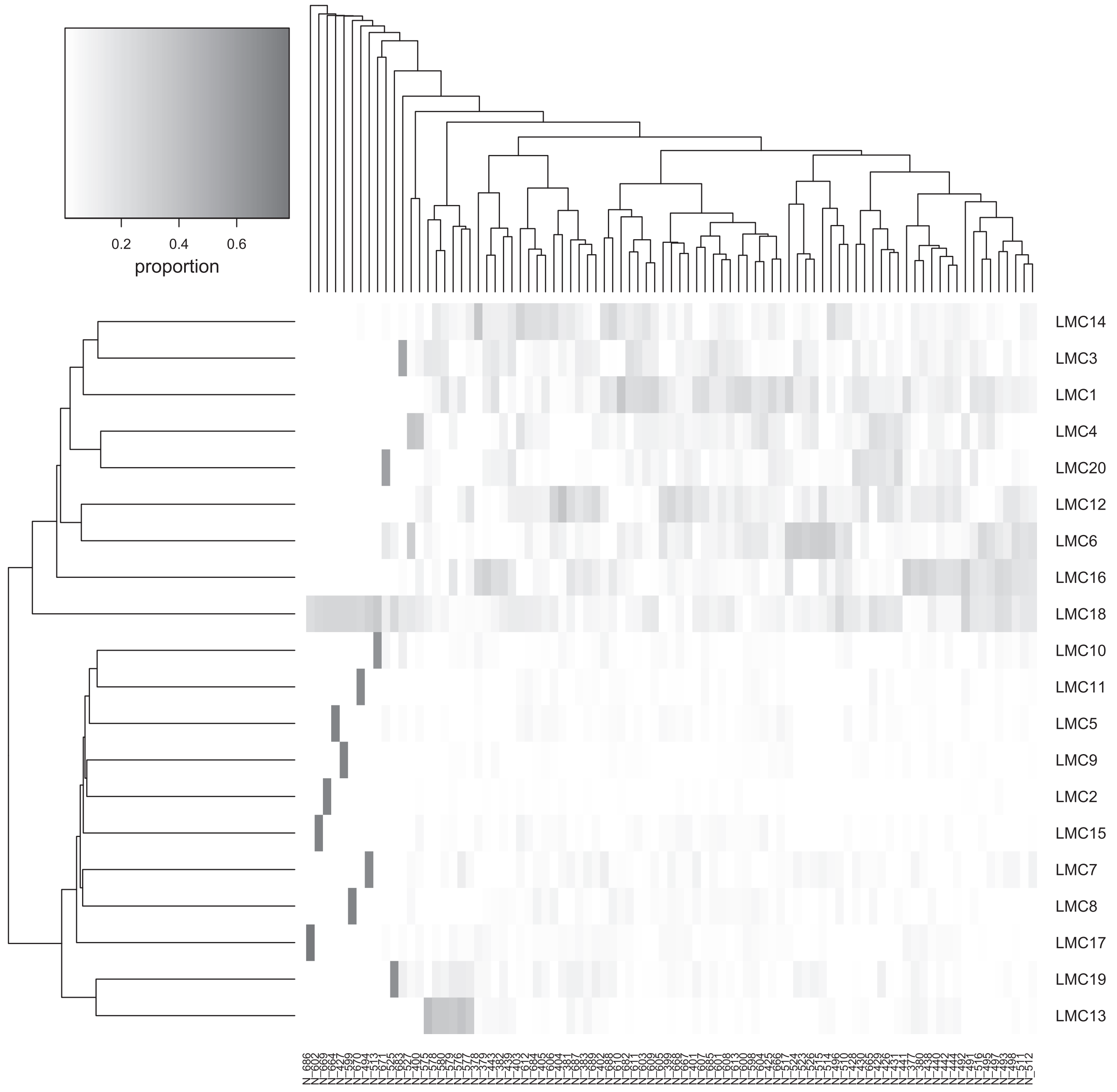

Figure S13: WB1 data set ($k = 20$, $\lambda = 0.001$), heat map of the recovered mixing proportions. Rows and columns were clustered to improve readability (hierarchical clustering with euclidean distance and average linkage).

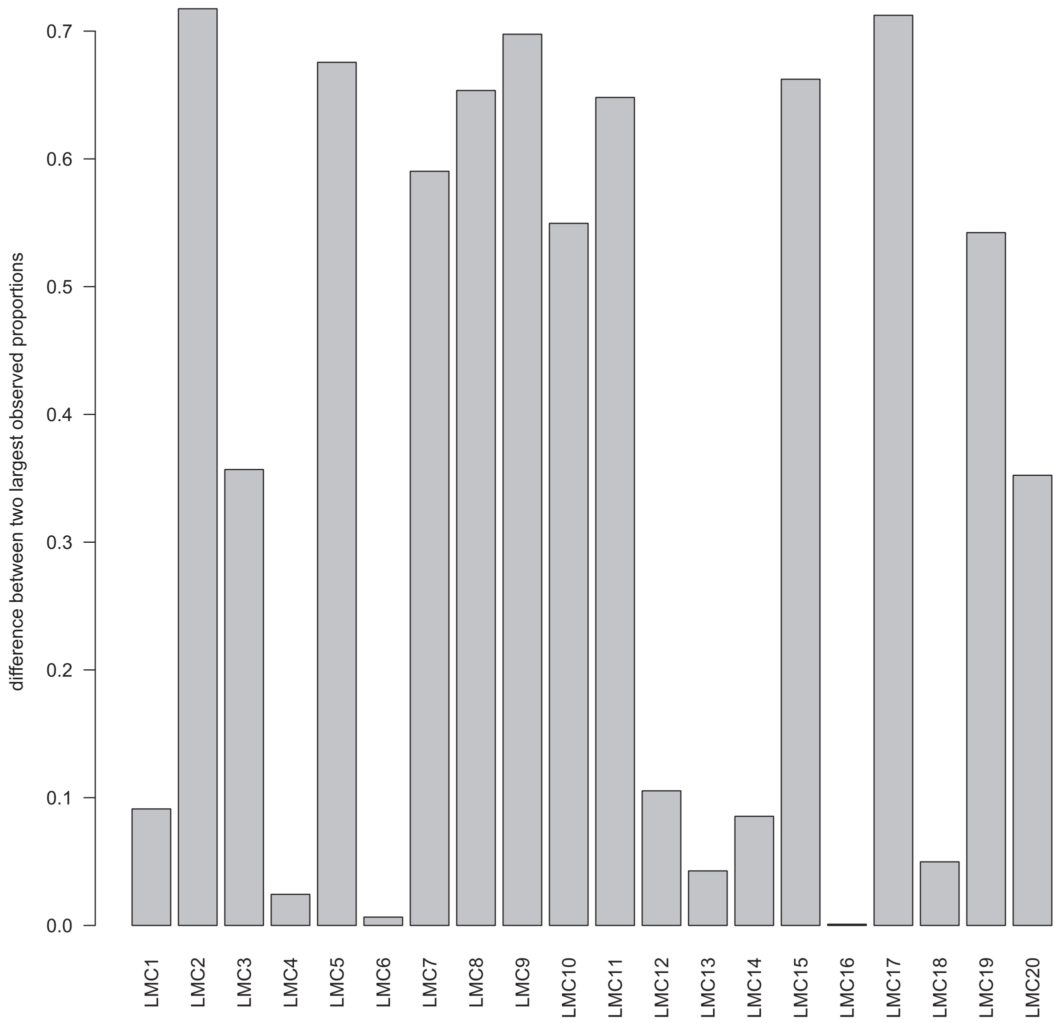

Figure S14: Individual-specific LMCs in the WB1 data set ($k = 20$, $\lambda = 0.001$). For each LMC a difference between the largest and the second largest observed proportion was calculated.

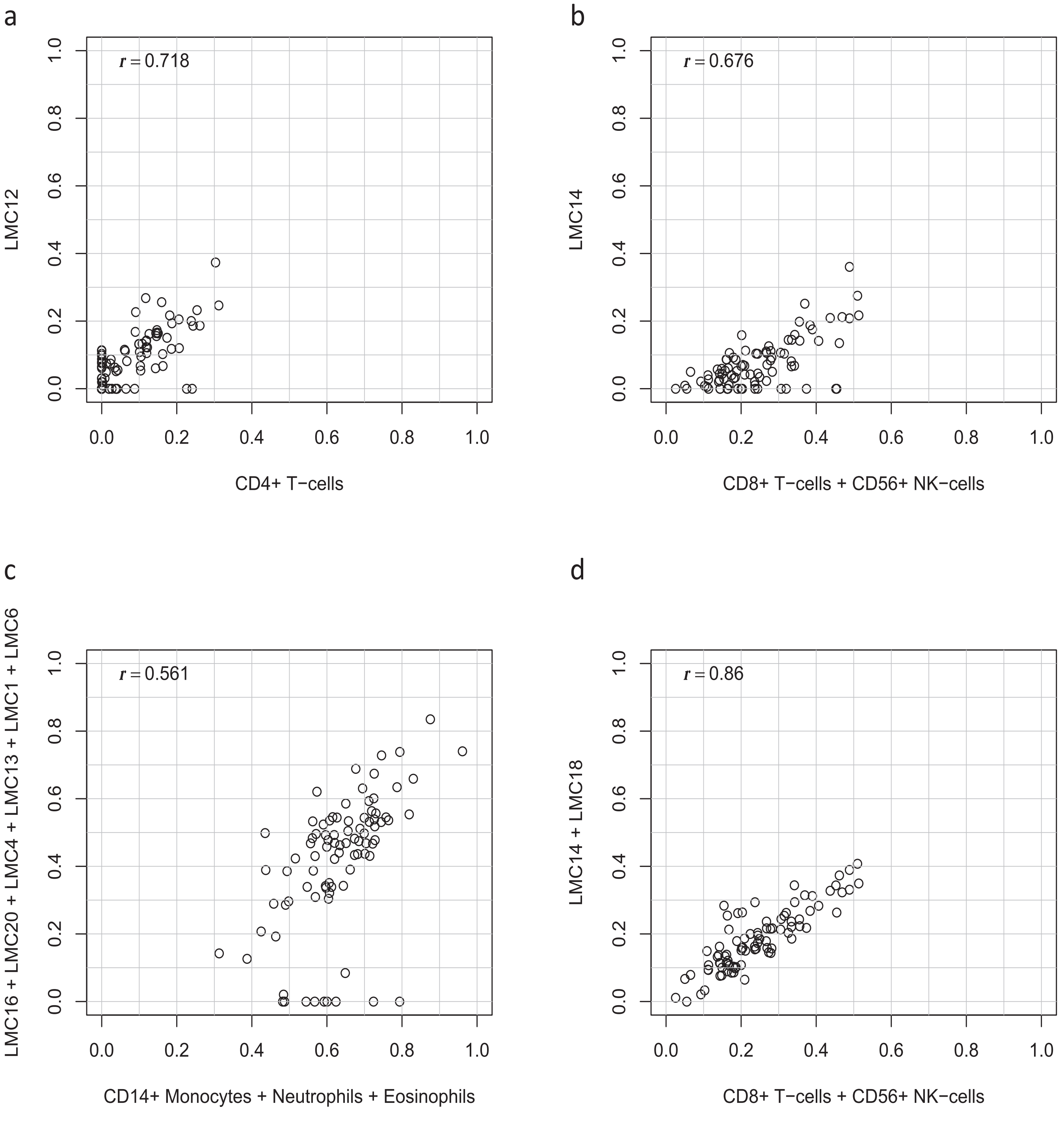

Figure S15: WB1 data set, proportion recovery. a. CD4+ T-cells. b. CD8+ T-cells and NK-cells. c. Myeloid cell types. Here individual-specific LMCs (see Figures S13 and S14) matching the "myeloid cluster" were excluded. d. CD8+ T-cells and NK-cells, with LMC18 included.

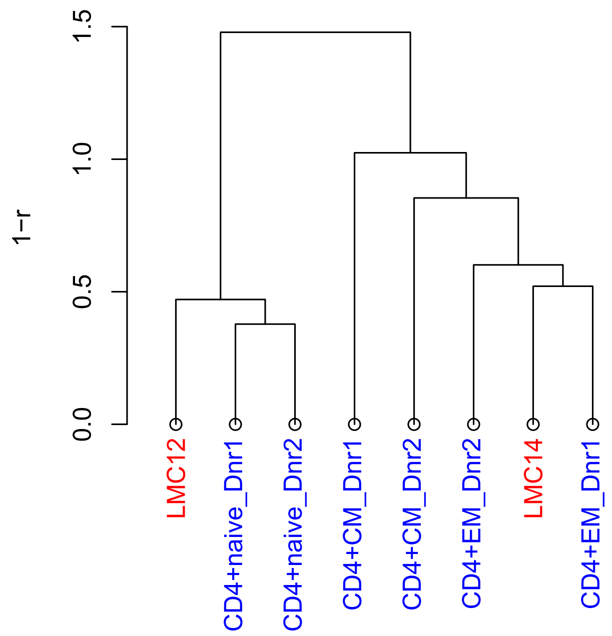

Figure S16: Matching the T-cell-specific LMCs from WB1 data set to reference WGBS-based CD4+ T-cell profiles. CD4+naive – CD4+ naive T-cells; CD4+CM – CD4+ central memory T-cells; CD4+EM – effector memory T-cells.

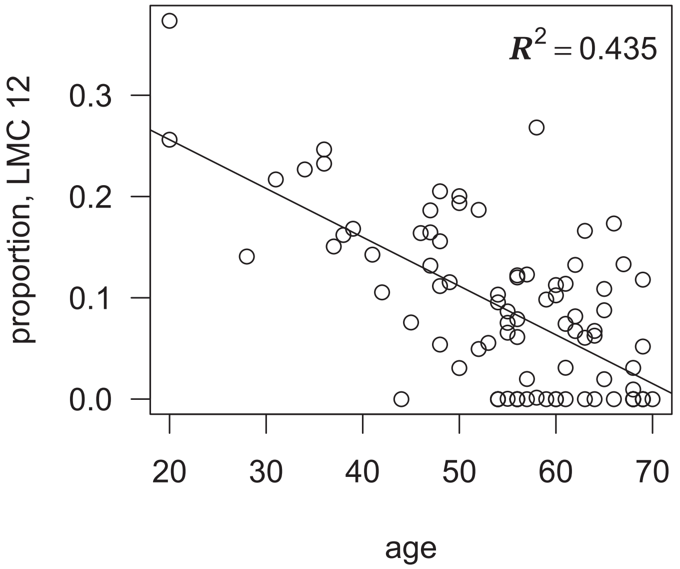

Figure S17: Proportion of LMC12 from WB1 correlates with the age of healthy individuals.

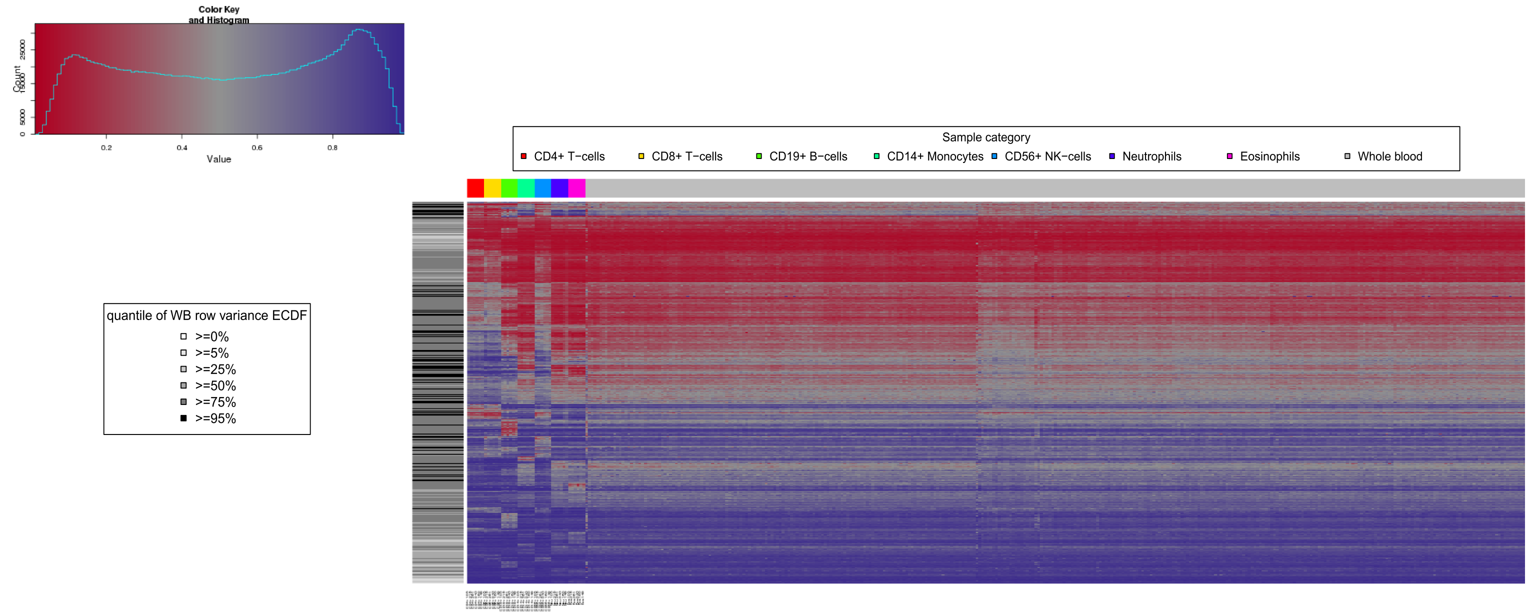

Figure S18: Preprocessed Infinium 450k methylation calls in PureBC and WB1 data at 15,000 CpGs with highest cell type specificity. The rows are ordered based on hierarchical clustering in the PureBC data only. The whole blood columns are ordered based on hierarchical clustering in the whole blood data only. The row color code reflects the quantile of empirical CpG-wise variance distribution in the whole blood data into which the variance of the corresponding CpG falls.

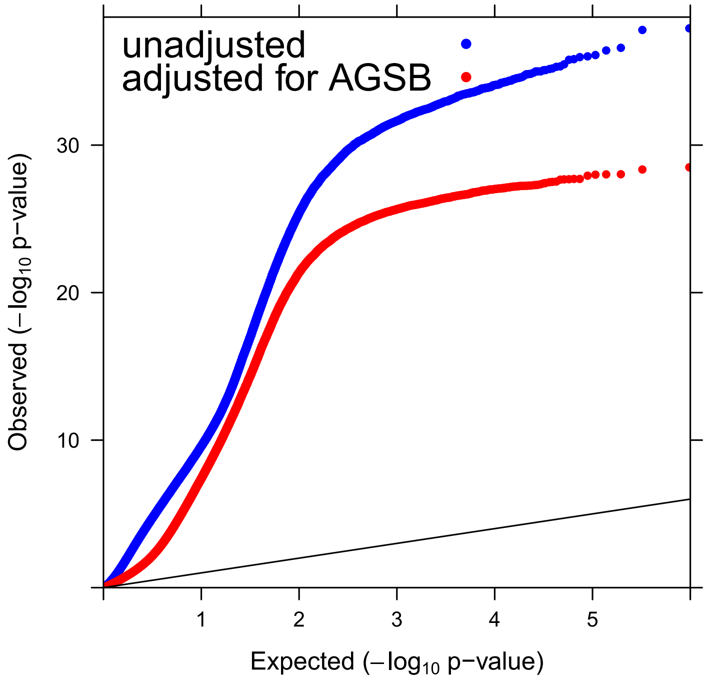

Figure S19: Inflation of significance in the association analysis for rheumatoid arthritis, full Liu *et al.* data set. AGSB, common covariates including age, gender, smoking status and experimental batch.

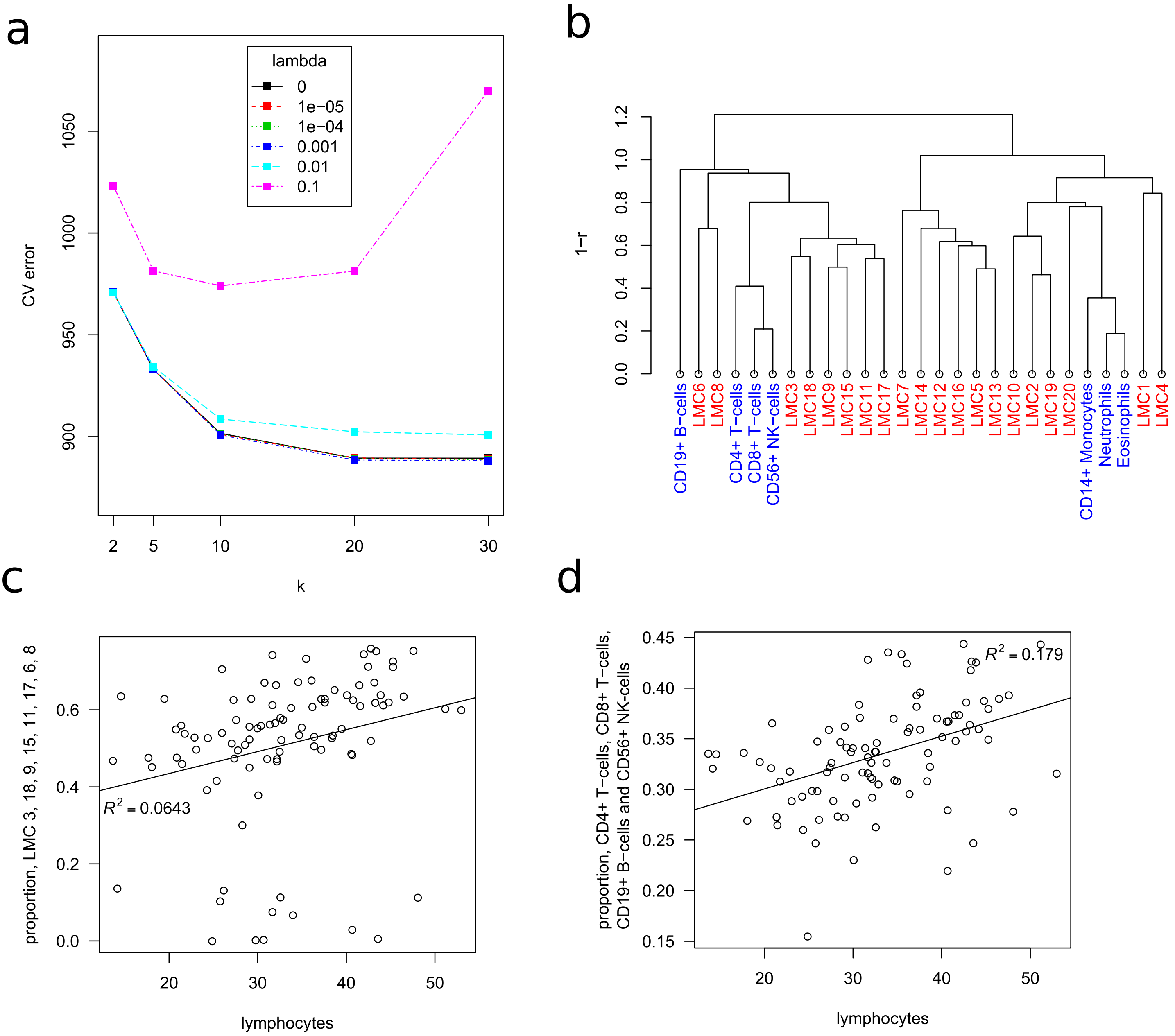

Figure S20: Decomposition of the whole blood profiles from the GALAII data set (Rahmani *et al.*). **a.** Parameter selection by cross-validation. **b.** Matching of recovered LMCs ($k = 20$, $\lambda = 5.0 \times 10^{-4}$). **c.** Summarized proportion of LMCs from the lymphoid cluster versus lymphocyte cell counts. **d.** Summarized proportion of the lymphoid cell types estimated by the reference-based method by Houseman *et al.* versus the lymphocyte cell counts.

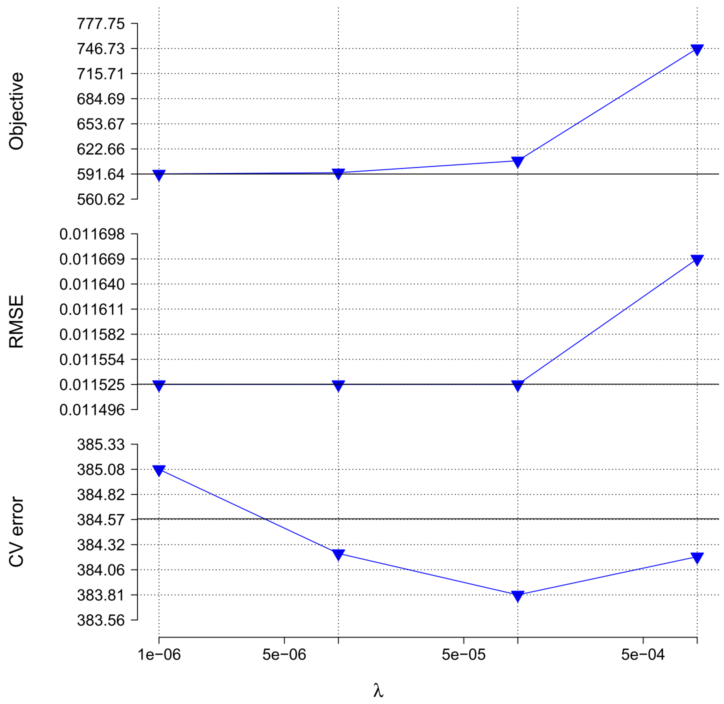

Figure S21: λ selection for the PureBC data set ($k = 16$)

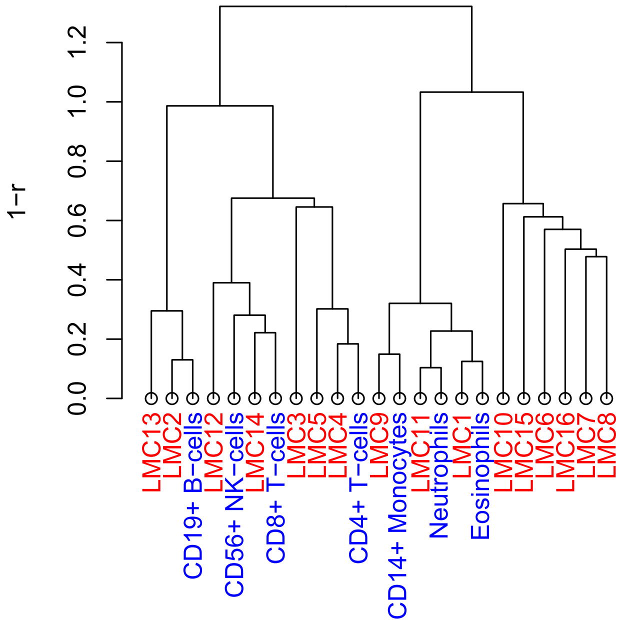

Figure S22: Matching of the LMCs from the PureBC data to average cell type profiles ($k = 16$, $\lambda = 10^{-4}$).

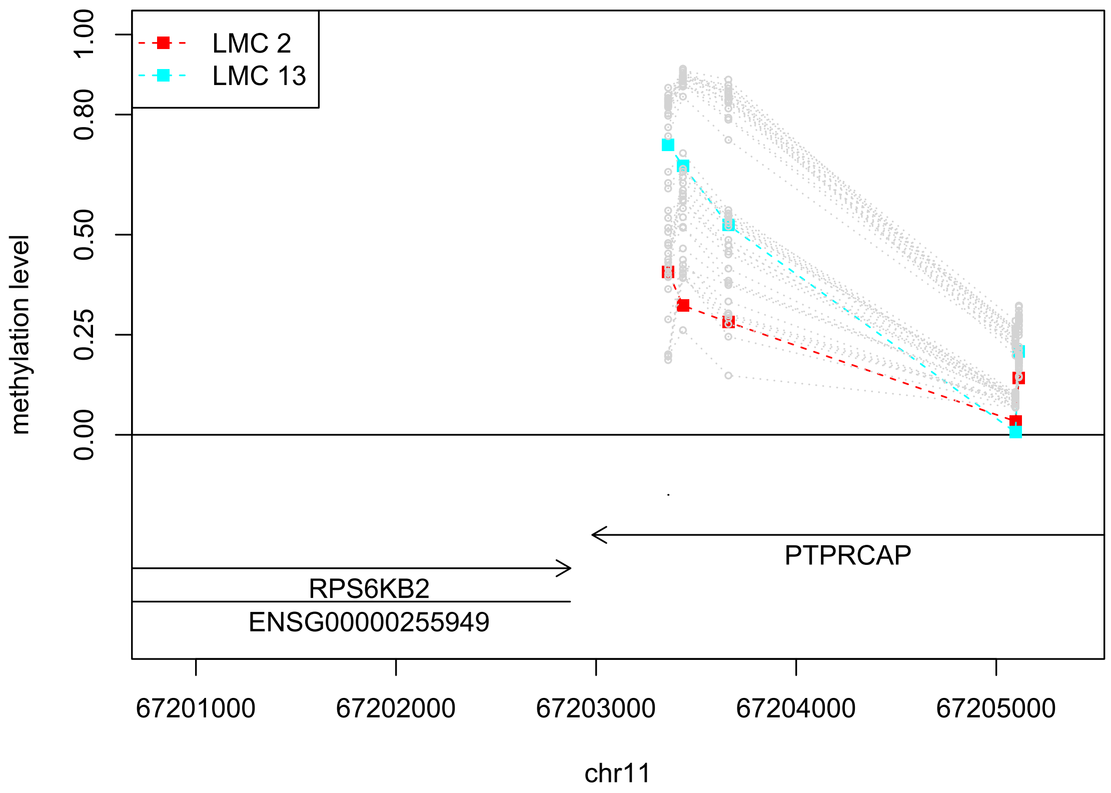

Figure S23: Purified blood cells: methylation level of the *PTPRCAP* locus in different purified blood cells (grey dotted lines), LMC2 and LMC13.

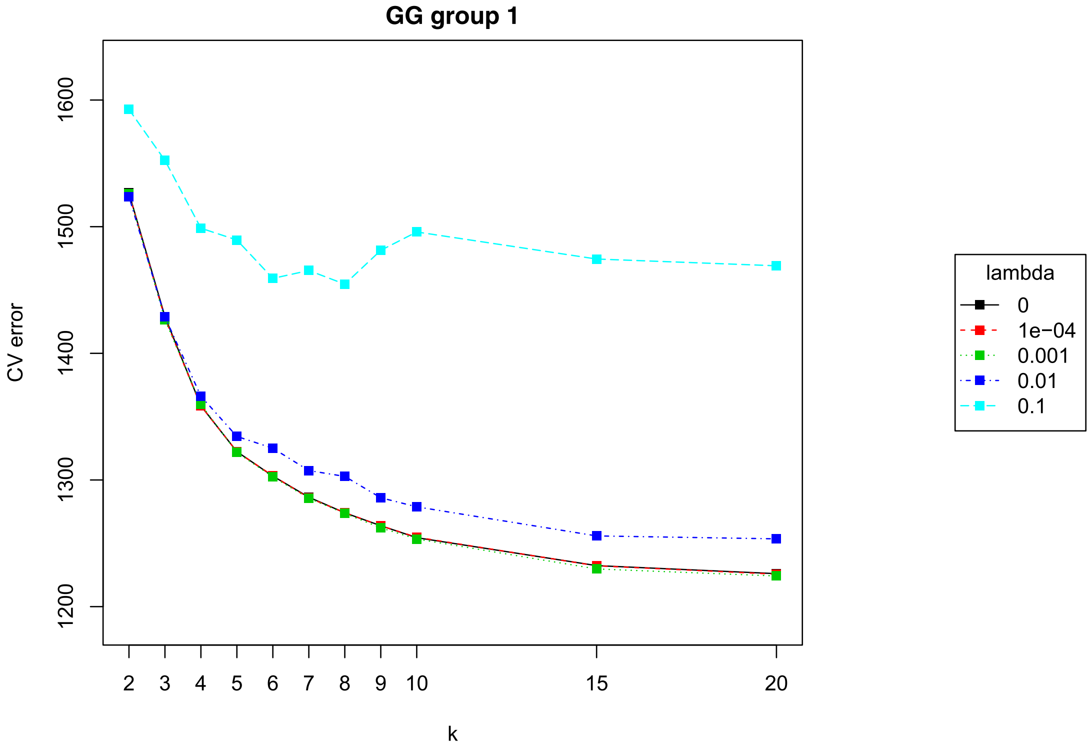

Figure S24: FC2 data set, parameter selection

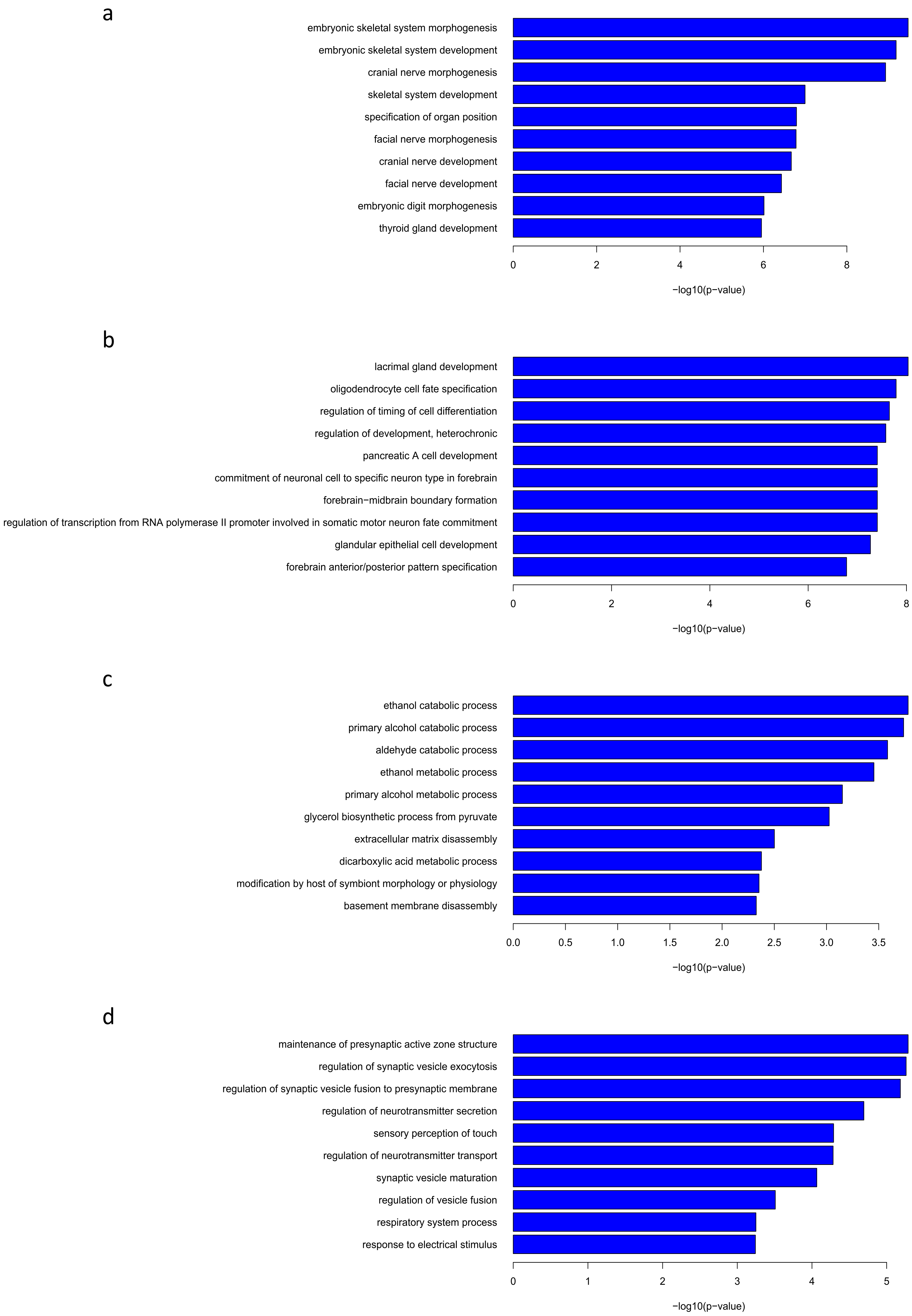

Figure S25: Functional annotation of frontal cortex LMCs. Raw (unadjusted) p-values of the GREAT binomial test are reported. a. LMC1, hypermethylated CpGs b. LMC1, hypomethylated CpGs. c. LMC2, hypermethylated CpGs d. LMC2, hypomethylated CpGs.

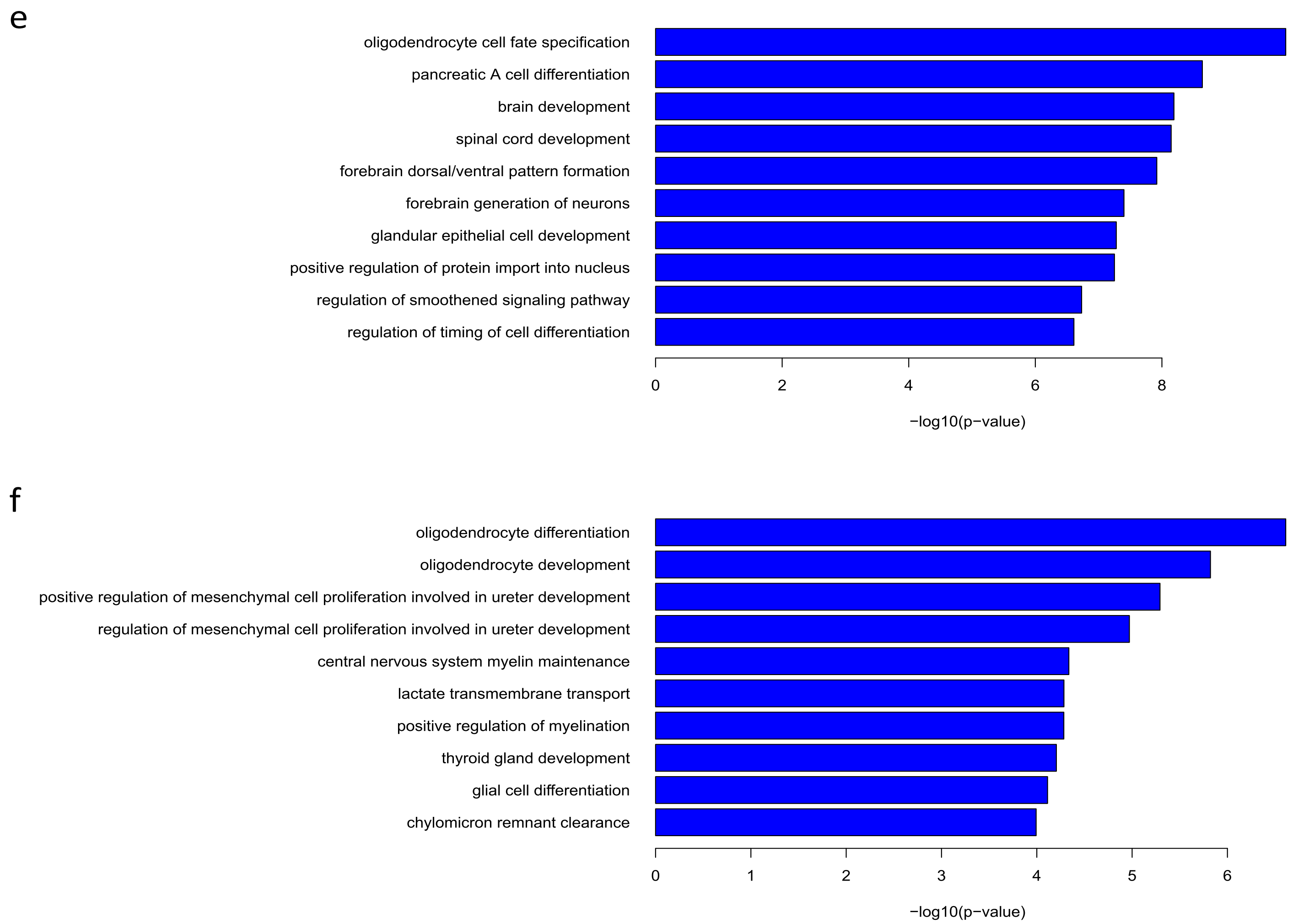

Figure S25: (continued) Functional annotation of frontal cortex LMCs. e. LMC3, hypermethylated CpGs. f. LMC3, hypomethylated CpGs.

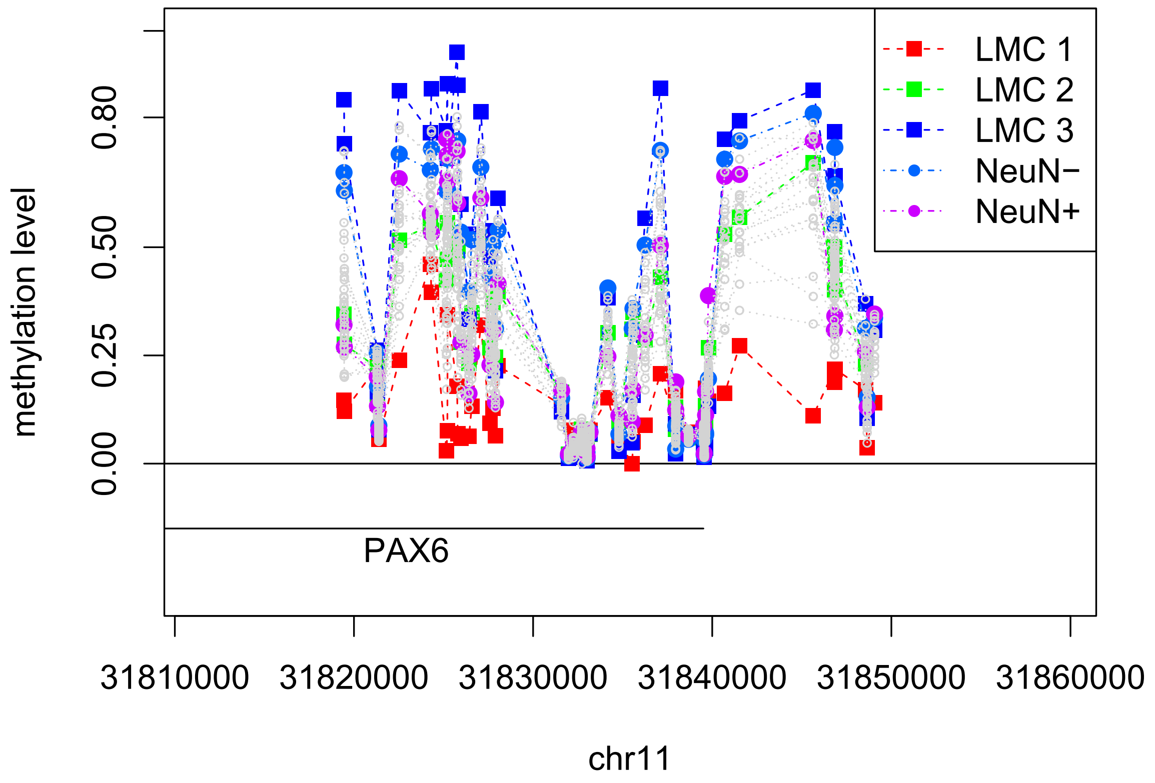

Figure S26: FC1 data set ($k = 3$, $\lambda = 0.003$), example of an LMC1-specific locus *PAX6*. Grey dotted lines correspond to the original frontal cortex profiles.

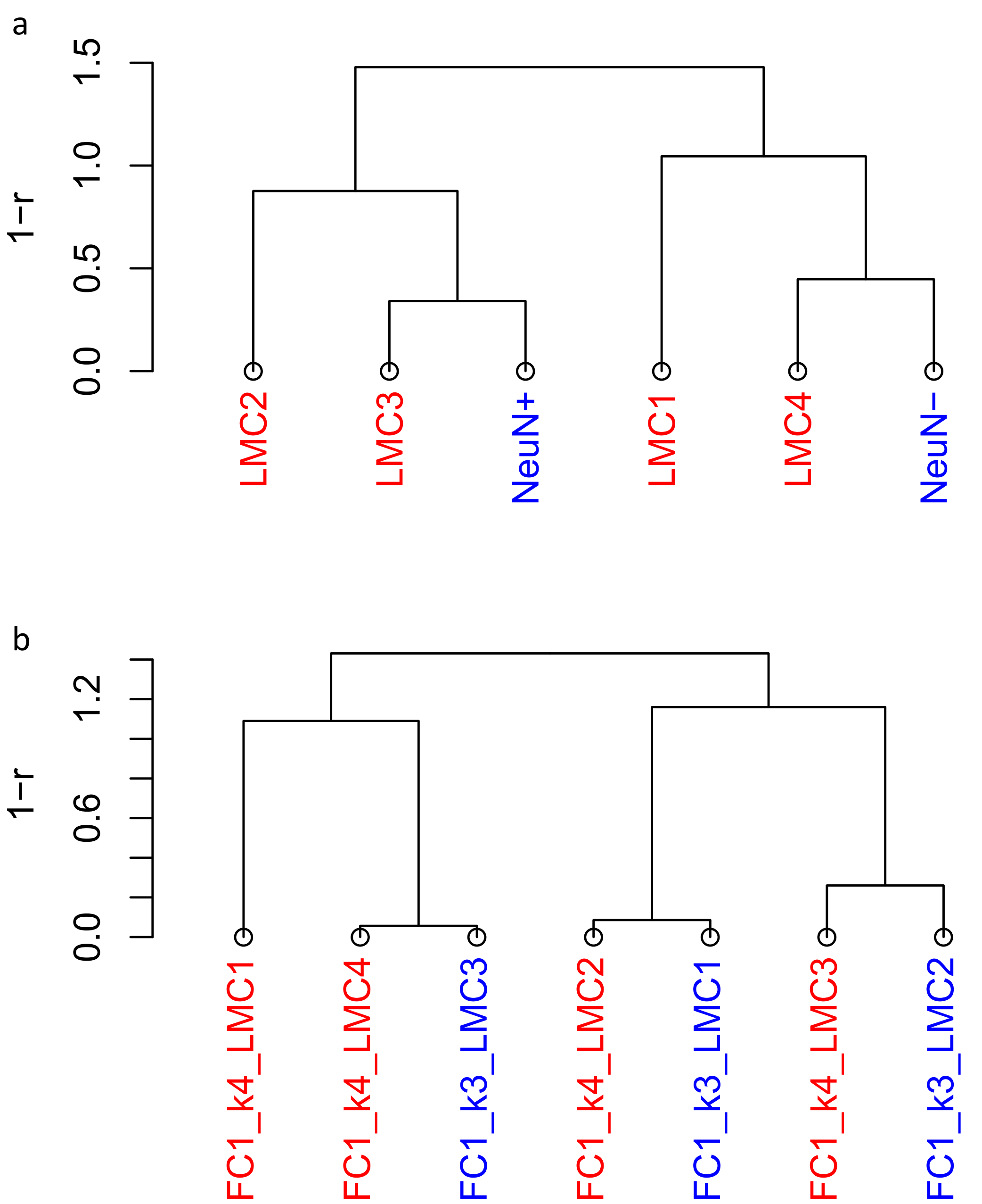

Figure S27: FC1 data set, MeDeCom solution with $k = 4$ and $\lambda = 0.005$ used for the proportion estimation. a. Matching LMCs to the PureN reference. b. Matching LMCs to the LMCs of the $k = 3$ case.

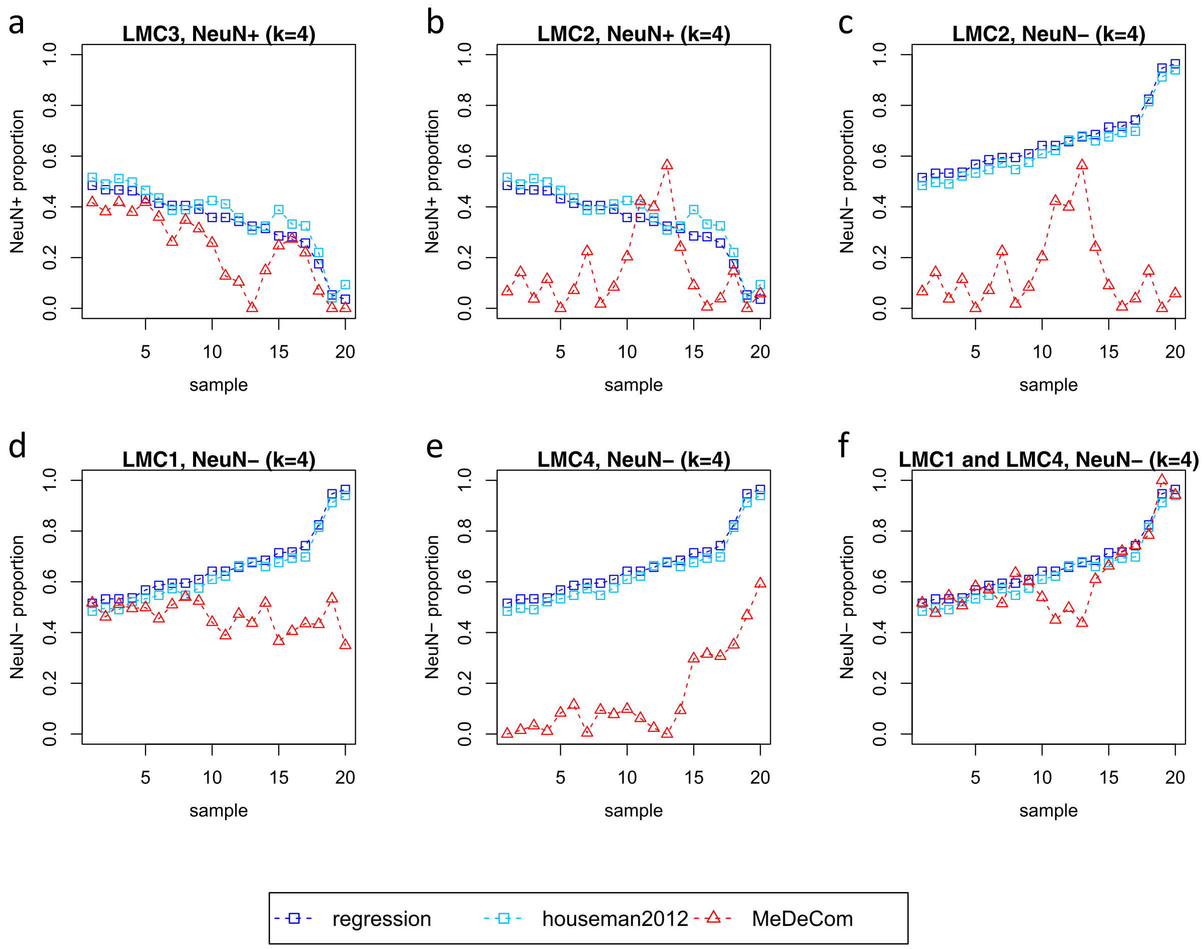

Figure S28: LMC contributions in comparison to the reference-based proportion estimates ($k = 4$, $\lambda = 5.0 \times 10^{-4}$). "Truth" stands for true mixing proportions, "regression" denotes the reference-based proportion estimation as described in the Methods. In each line plot the samples are sorted by ascending reference-based proportion estimates. In **f** the proportions are summarized for LMC1 and LMC4.

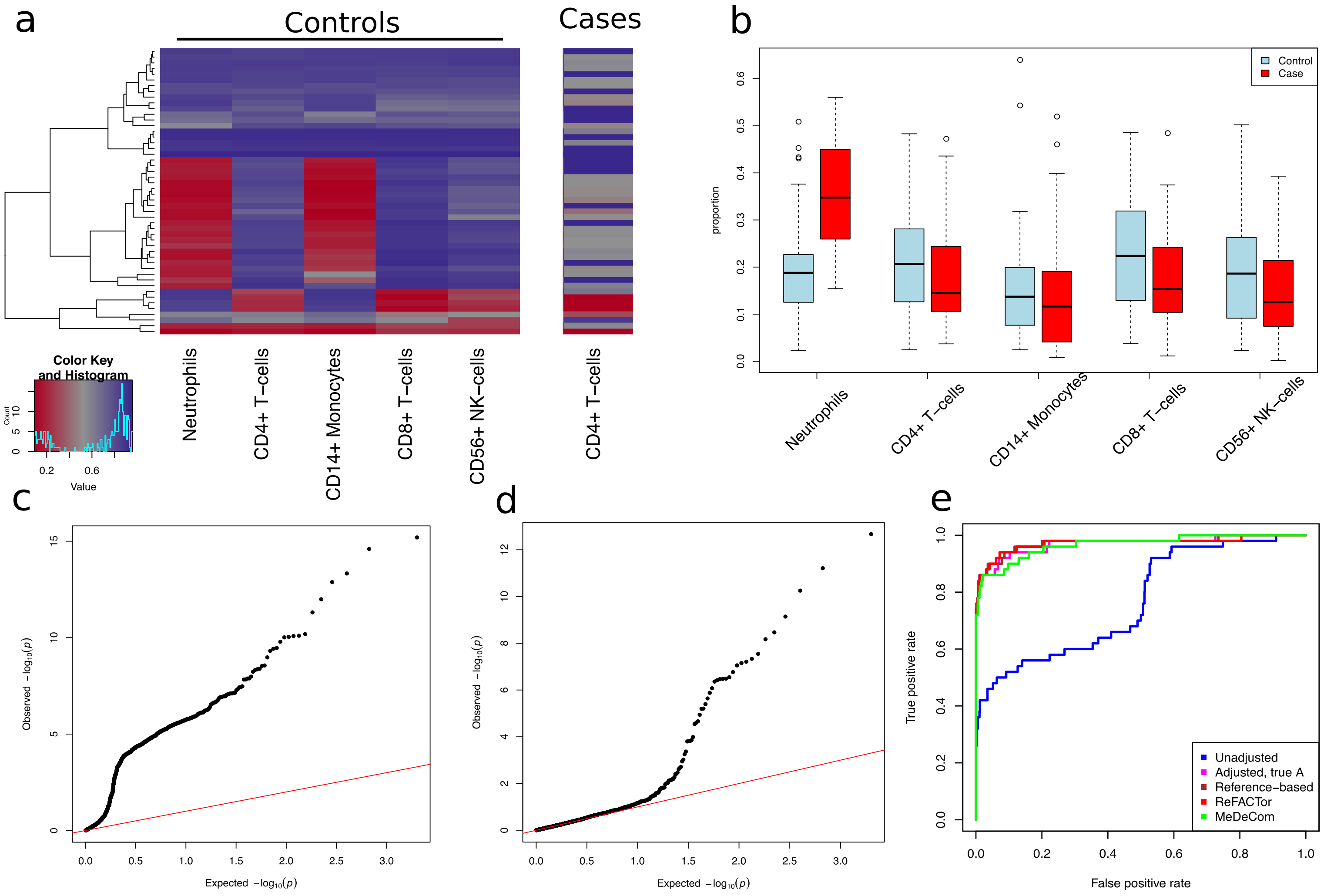

Figure S29: Application of MeDeCom to a synthetic example with simulated true methylation change in one cell type and confounding by cell type proportions. **a.** Methylation effects with effect sizes sampled from $\mathcal{N}(0.25, 0.05)$ were introduced into the CD4+ T-cell profile at 50 (shown) of 1000 total CpGs with a random sign. **b.** Confounding was added by increasing the unscaled parameter of the Dirichlet distribution (see Simulation) controlling the proportion of the Neutrophils. **c.** P-values of the unadjusted association analysis (CpG-wise T-test) show signs of strong inflation. **d.** Adjustment using LMC proportions decreases the inflation and moderates the association analysis. **e.** Systematic comparison of all adjustment methods using ROC-curves shows similar performance.

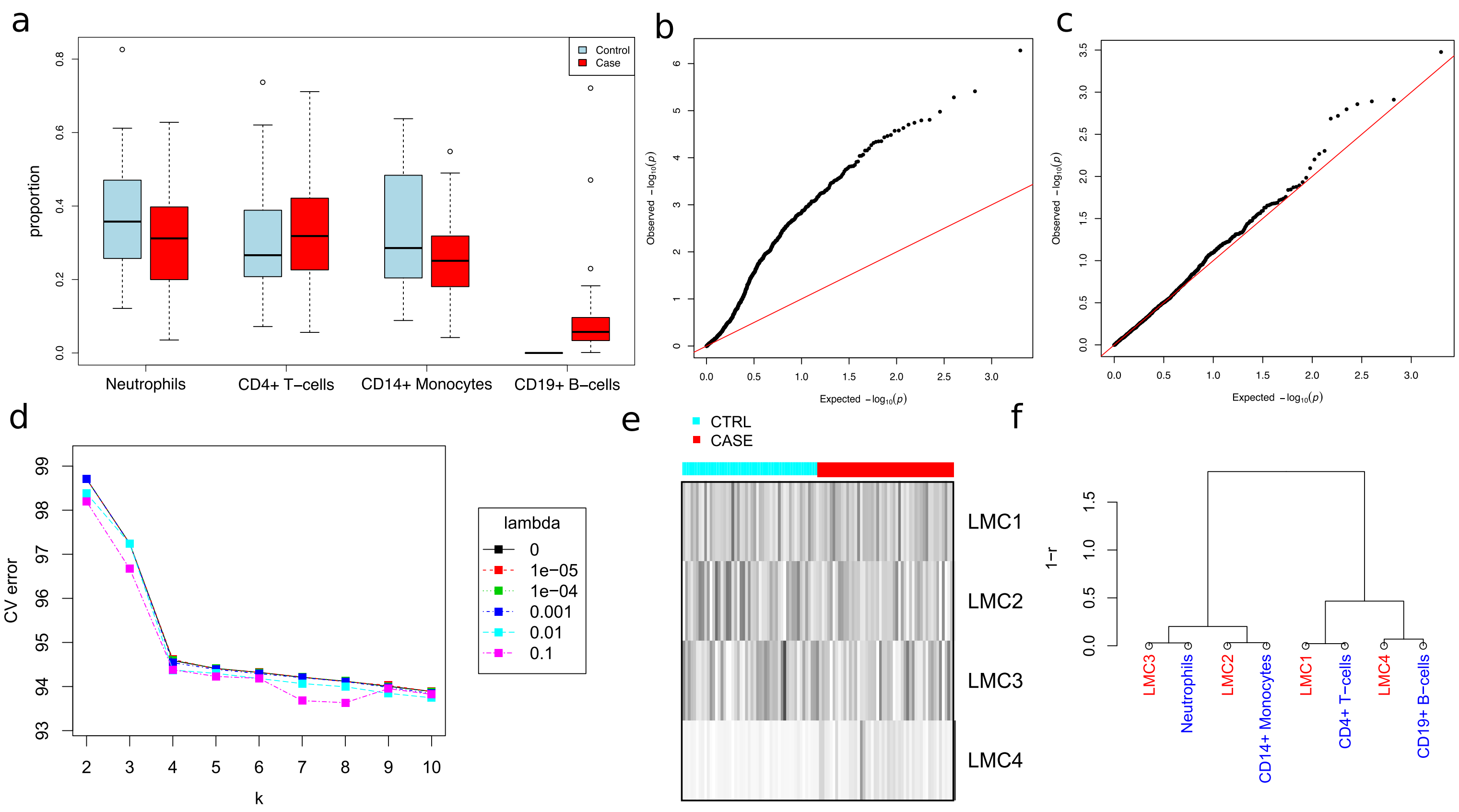

Figure S30: Application of MeDeCom to a synthetic example with a rare cell populations present in the cases as the only true effect. **a.** Both cases and controls contain roughly equal amounts of 3 abundant cell types. A small admixture of B-cells in the cases is the true effect of interest. b. Standard unadjusted analysis reveals moderate inflation for the majority of CpGs. **c.** After adjustment with ReFACTor the inflation is eliminated and no significant associations are detected (not shown). **d.** MeDeCom detects the presence of four LMCs. **e.** and **f.** Visualisation of the recovered proportions reveals that LMC4 is enriched only in the cases. **g.** Recovered LMCs accurately reproduce the source cell type methylomes, in particular LMC4 is corresponding to B-cells.

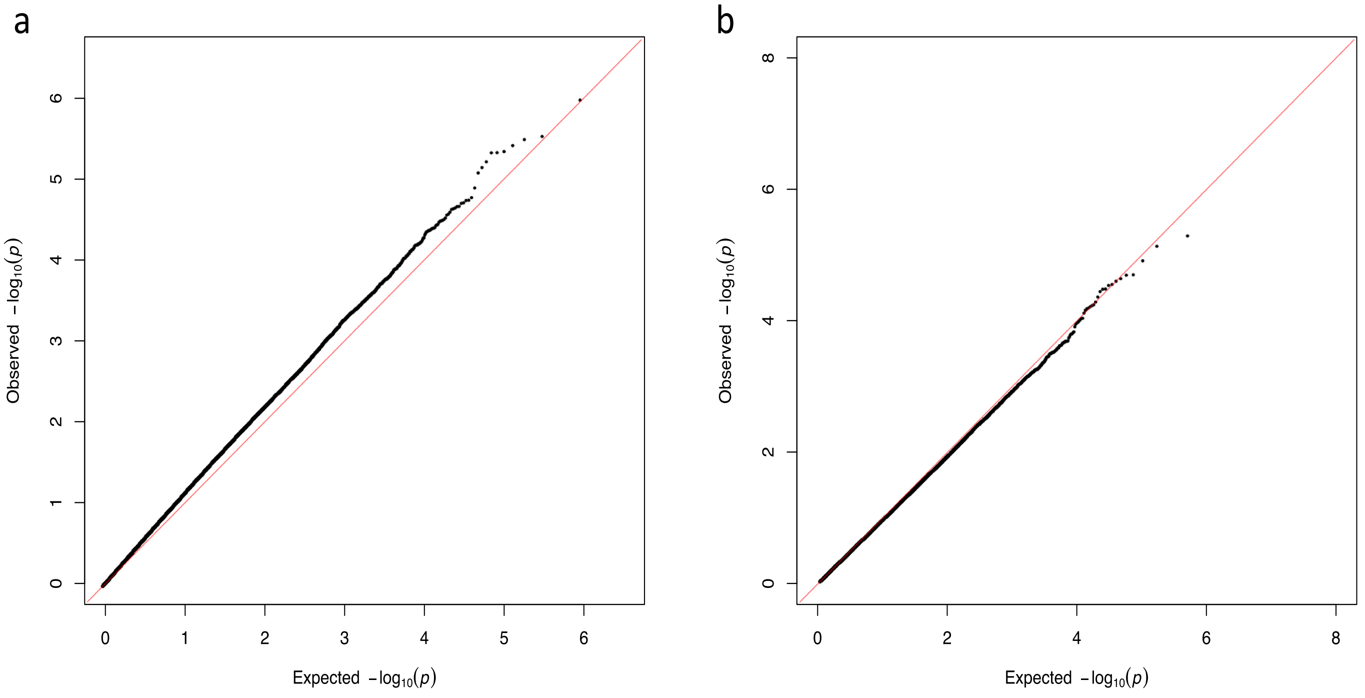

Figure S31: Association analysis for the AD phenotype in the FC2 data set. **a.** P-value Q-Q plot of the association analysis for Braak stage with adjustment for sex and age. **b.** Same as **a.** with an additional adjustment for 3 LMCs (1,2, and 8) having the strongest association with the Braak stage.

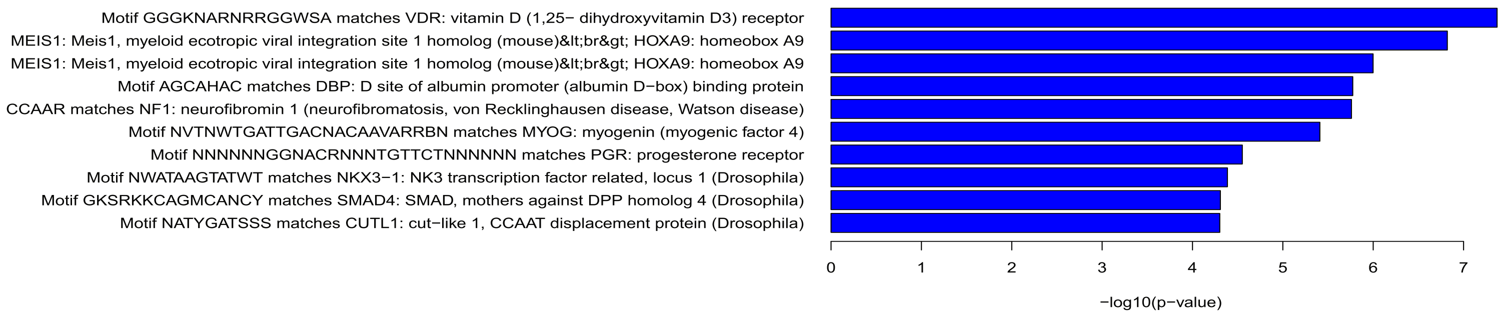

Figure S32: Enrichment of the transcription factor binding sites in the promoters of the LMC2-associated (hyper-methylated) genes.

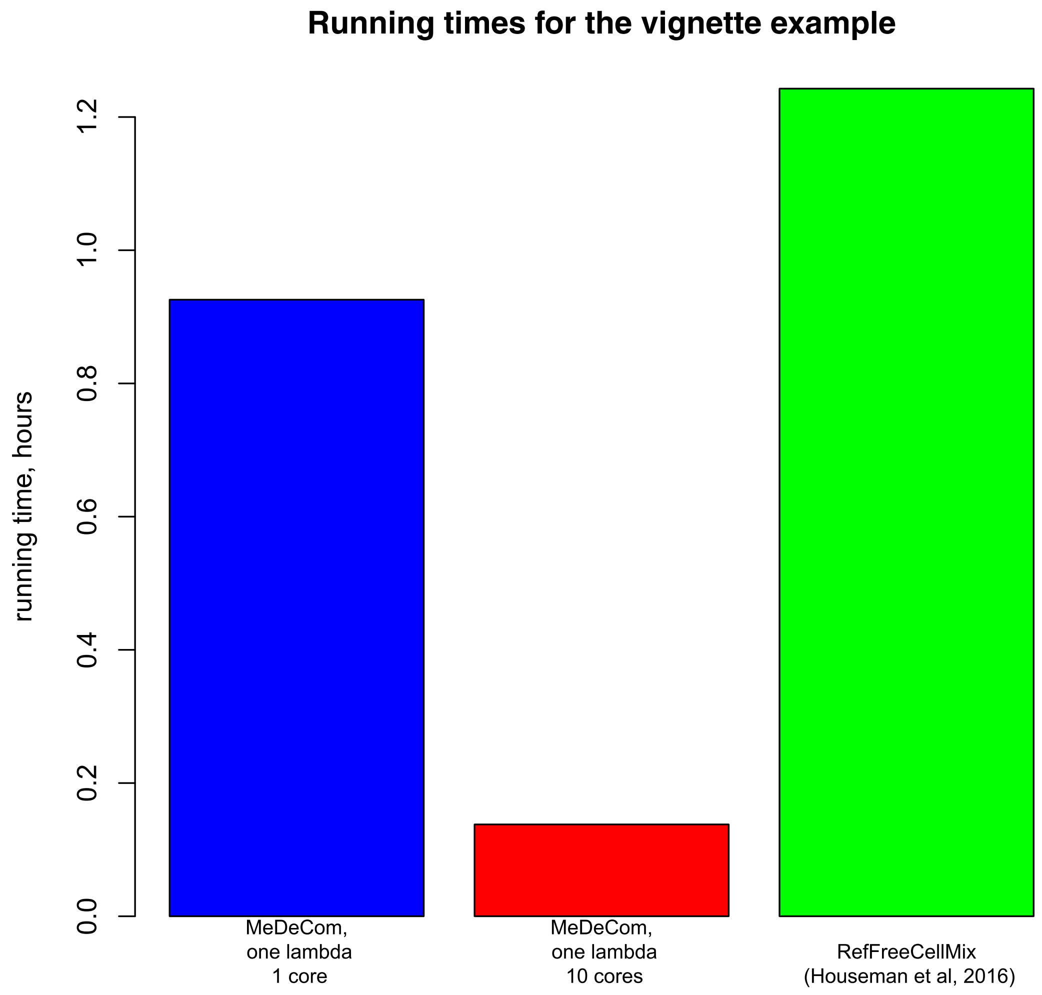

Figure S33: Running time comparison between MeDeCom and RefFreeCellMix. Both methods were applied to the example data set from the MedeCom vignette. To make the computational burden comparable, we used only one value of the regularization parameter ($\lambda = 0$). Maximal number of iterations was set to 300 in both cases. The tests were performed on one compute node (32 cores and 128 GB RAM) in a single machine mode.
