# Supplementary material for: MeDeCom: discovery and quantification of latent components of heterogeneous methylomes
Source: Genome Biol. 2017 Mar 24;18:55. doi: 10.1186/s13059-017-1182-6 (PMC5366155; doi:10.1186/s13059-017-1182-6)
Supplement: Supplementary file 5 — Supplementary Text. PDF document with supplementary notes. (PDF 158 kb) [file 13059_2017_1182_MOESM5_ESM.pdf]

1. "Exact" and "approximate" models for heterogeneous DNA methylation profiles

Model definition

Given the negligible measurement error, the methylation data $\mathbf{D}$ at m CpG positions from multi-cellular samples of n individuals can be represented as:

$$\mathbf{D} = \mathbf{CF} \tag{S.1}$$

Here, $\mathbf{C}$ is an $m \times q$ matrix of all single-cell DNA methylation profiles existing in all cell populations of all individuals, and $\mathbf{F}$ is an $m \times q$ matrix representing the frequency of given single cell profile in a measured sample.

Theoretical upper bound for q, given the three possible DNA methylation states in the cell $\{0, 0.5, 1\}$, is 3^m. However, the vast majority of potentially possible DNA methylation profiles are not biologically feasible, and $q \ll 3^m$. Nevertheless, in real applications q and m have comparable order of magnitude, and, quite certainly, $q \gg n$. The latter fact makes it impossible to find $\mathbf{C}$ and $\mathbf{F}$ computationally.

Matrix $\mathbf{C}$ is expected to have a complicated correlation structure. Clustering of its columns should reveal multiple nested groups of single cell DNA methylation patterns, reflecting the hierarchy of cell types and subtypes. One may than fix k as a number of discrete cell populations with highly similar DNA methylation profiles, depending on a certain similarity threshold. Given a set $\mathcal{C}_s$ of matrix $\mathbf{C}$ columns which represents the s-th such population, one could decrease the largest dimension in eq. (1), by constructing a summary profile $\mathbf{t}_s$ of such population: $\mathbf{t}_s = \frac{1}{n}\mathbf{C}_{:,\mathcal{C}_s}\mathbf{F}_{\mathcal{C}_s,:}\mathbf{W}^{-1}\mathbf{1}_n$, where $\mathbf{W}$ is diagonal matrix with $\mathrm{diag}(\mathbf{W}) = \mathbf{F}^T_{\mathcal{C}_s,:}\mathbf{1}_{|\mathcal{C}_s|}$, and then approximating the measured DNA methylation data as:

$$\mathbf{D} \approx \mathbf{TA} \tag{S.2}$$

where $\mathbf{a_s} = \mathbf{1}^T_{|C_s|}\mathbf{F}_{\mathcal{C}_s,:}$. In the extreme case of all the columns in $\mathbf{C}_{:,\mathcal{C}_s}$ being identical profile $\mathbf{t}_s$ is obviously also identical to any of these and the approximate model (2) holds exactly. In fact this could be enforced by selecting a subset of rows $\mathcal{R}_s$, such that all columns of $C_{\mathcal{R}_s,\mathcal{C}_s}$ are identical (cell type-specific marker selection). Then the model in (2) augmented to the intersect of all such subsets $\mathcal{R}_s$, $s = 1 \ldots k$, would also hold exactly (see subsection below).

In case $k \leq n$, i.e. the selected number of discrete populations is comparable to the number of profiled individuals, both $\mathbf{T}$ and $\mathbf{A}$ can in theory be recovered computationally.

The approximation error

One can estimate the error of this approximation. In case only one the s-th pattern set is substituted, the error is

$$\begin{aligned} err_s &= \left\|\mathbf{t}_s\mathbf{a}_s - \mathbf{C}_{:,C_s}\mathbf{F}_{C_s,:}\right\|_2 = \\ = &\quad \left\|\frac{1}{n}\mathbf{C}_{:,\mathcal{C}_s}\mathbf{F}_{\mathcal{C}_s,:}\mathbf{W}^{-1}\mathbf{1}_n\mathbf{1}^T_{|C_s|}\mathbf{F} - \mathbf{C}_{:,C_s}\mathbf{F}_{C_s,:}\right\|_2 = \\ &= \left\|\mathbf{C}_{:,\mathcal{C}_s}\left(\frac{1}{n}\mathbf{F}_{\mathcal{C}_s,:}\mathbf{W}^{-1}\mathbf{1}_n\mathbf{1}^T_{|\mathcal{C}_s|} - \mathbf{I}_{|\mathcal{C}_s|}\right)\mathbf{F}_{C_s,:}\right\|_2 = \left\|\mathbf{C}_{:,\mathcal{C}_s}\Delta^F_{|\mathcal{C}_s|,:}\right\|_2 \end{aligned}$$

One can show that r, j-th element of matrix $\mathbf{\Delta}^F$ represents the deviation of the r-th pattern frequency from the expected frequency based on the average across all individuals:

$$\boldsymbol{\Delta}^F_{\mathcal{C}_s} = \left(\frac{1}{n}\mathbf{F}_{\mathcal{C}_s,:}\mathbf{W}^{-1}\mathbf{1}_n\mathbf{1}^T_{|\mathcal{C}_s|} - \mathbf{I}_{|\mathcal{C}_s|}\right)\mathbf{F}_{\mathcal{C}_s,:} =$$

$$= \left(\begin{vmatrix} \frac{1}{n}\sum_{j'}\frac{f_{1,j'}}{\sum_{r'} f_{r',j'}} & \cdots & \frac{1}{n}\sum_{j'}\frac{f_{1,j'}}{\sum_{r'} f_{r',j'}} \\ \vdots & \ddots & \vdots \\ \frac{1}{n}\sum_{j'}\frac{f_{|\mathcal{C}_s|,j'}}{\sum_{r'} f_{r',j'}} & \cdots & \frac{1}{n}\sum_{j'}\frac{f_{|\mathcal{C}_s|,j'}}{\sum_{r'} f_{r',j'}} \end{vmatrix} - \mathbf{I}_{|\mathcal{C}_s|}\right)\mathbf{F}_{\mathcal{C}_s,:} =$$

$$= \begin{vmatrix} \frac{1}{n}\sum_{j'}\frac{f_{1,j'}}{\sum_{r'} f_{r',j'}} - 1 & \cdots & \frac{1}{n}\sum_{j'}\frac{f_{1,j'}}{\sum_{r'} f_{r',j'}} & \cdots & \frac{1}{n}\sum_{j'}\frac{f_{1,j'}}{\sum_{r'} f_{r',j'}} \\ \vdots & & \vdots & & \vdots \\ \frac{1}{n}\sum_{j'}\frac{f_{r,j'}}{\sum_{r'} f_{r',j'}} & \cdots & \frac{1}{n}\sum_{j'}\frac{f_{r,j'}}{\sum_{r'} f_{r',j'}} - 1 & \cdots & \frac{1}{n}\sum_{j'}\frac{f_{1,j'}}{\sum_{r'} f_{r',j'}} \\ \vdots & & \vdots & & \vdots \\ \frac{1}{n}\sum_{j'}\frac{f_{|\mathcal{C}_s|,j'}}{\sum_{r'} f_{r',j'}} & \cdots & \frac{1}{n}\sum_{j'}\frac{f_{|\mathcal{C}_s|,j'}}{\sum_{r'} f_{r',j'}} & \cdots & \frac{1}{n}\sum_{j'}\frac{f_{|\mathcal{C}_s|,j'}}{\sum_{r'} f_{r',j'}} - 1 \end{vmatrix}\mathbf{F}_{\mathcal{C}_s,:} =$$

$$= \begin{vmatrix} \frac{1}{n}\sum_{j'}\frac{f_{1,j'}}{\sum_{r'} f_{r',j'}}\cdot\sum_{r''} f_{r'',1} - f_{1,1} & \cdots & \frac{1}{n}\sum_{j'}\frac{f_{1,j'}}{\sum_{r'} f_{r',j'}}\cdot\sum_{r''} f_{r'',j} - f_{1,j} & \cdots & \frac{1}{n}\sum_{j'}\frac{f_{1,j'}}{\sum_{r'} f_{r',j'}}\cdot\sum_{r''} f_{r'',n} - f_{1,n} \\ \vdots & \ddots & \vdots & \ddots & \vdots \\ \frac{1}{n}\sum_{j'}\frac{f_{r,j'}}{\sum_{r'} f_{r',j'}}\cdot\sum_{r''} f_{r'',1} - f_{r,1} & \cdots & \frac{1}{n}\sum_{j'}\frac{f_{r,j'}}{\sum_{r'} f_{r',j'}}\cdot\sum_{r''} f_{r'',j} - f_{r,j} & \cdots & \frac{1}{n}\sum_{j'}\frac{f_{r,j'}}{\sum_{r'} f_{r',j'}}\cdot\sum_{r''} f_{r'',j} - f_{r,n} \\ \vdots & \ddots & \vdots & \ddots & \vdots \\ \frac{1}{n}\sum_{j'}\frac{f_{|\mathcal{C}_s|,j'}}{\sum_{r'} f_{r',j'}}\cdot\sum_{r''} f_{r'',1} - f_{|\mathcal{C}_s|,1} & \cdots & \frac{1}{n}\sum_{j'}\frac{f_{|\mathcal{C}_s|,j'}}{\sum_{r'} f_{r',j'}}\cdot\sum_{r''} f_{r'',n} - f_{|\mathcal{C}_s|,j} & \cdots & \frac{1}{n}\sum_{j'}\frac{f_{|\mathcal{C}_s|,j'}}{\sum_{r'} f_{r',j'}}\cdot\sum_{r''} f_{r'',n} - f_{|\mathcal{C}_s|,n} \end{vmatrix}$$

Here the term $\frac{1}{n}\sum_{j'}\frac{f_{r,j'}}{\sum_{r'} f_{r',j'}}\cdot\sum\limits_{r} f_{r,j}$ represents the expected frequency of pattern r in the individual j, based on the average in all individuals, and each element of $\boldsymbol{\Delta}^F_{\mathcal{C}_s}$ reflects the deviation of the actually observed frequency from this average value.

It is now easy to see why err_s is zero in case all the patterns in $\mathbf{C}_{:,\mathcal{C}_s}$ are identical. Consider one element of $\mathbf{C}_{:,\mathcal{C}_s}\Delta^F_{|\mathcal{C}_s|,:}$:

$$(\mathbf{C}_{:,\mathcal{C}_s}\Delta^F_{|\mathcal{C}_s|,:})_{i,j} = \sum_r \left(\frac{1}{n}\sum_{j'}\frac{f_{r,j'}}{\sum_{r'} f_{r',j'}}\cdot\sum_r f_{r,j} - f_{r,j}\right) c_{i,r}$$

The most trivial case is when $c_{i,r} = 0,\ r = 1 \ldots |\mathcal{C}_s|$, i.e. the CpG position i is unmethylated in all patterns from $\mathcal{C}_s$. In case this CpG position is fully methylated in all patterns ($c_{i,r} = 1,\ r = 1 \ldots |\mathcal{C}_s|$):

$$\sum_r \left(\frac{1}{n}\sum_{j'}\frac{f_{r,j'}}{\sum_{r'} f_{r',j'}}\cdot\sum_r f_{r,j} - f_{r,j}\right) c_{i,r} = \sum_r \left(\frac{1}{n}\sum_{j'}\frac{f_{r,j'}}{\sum_{r'} f_{r',j'}}\cdot\sum_r f_{r,j} - f_{r,j}\right) =$$

$$= \sum_r \left(\frac{1}{n}\sum_{j'}\frac{f_{r,j'}}{\sum_{r'} f_{r',j'}}\cdot\sum_r f_{r,j}\right) - \sum_r f_{r,j} = \frac{1}{n}\left(\sum_{j'}\frac{\sum_r f_{r,j'}}{\sum_{r'} f_{r',j'}}\right)\cdot\sum_r f_{r,j} - \sum_r f_{r,j} = \frac{1}{n}\cdot n\cdot\sum_r f_{r,j} - \sum_r f_{r,j} = 0$$

In all other cases, i.e. non-identical methylation levels across $c_{i,r}, r = 1 \ldots |\mathcal{C}_s|$, the approximation error $err_s \neq 0$.

2. Including prior information

Model fitting as outlined in the Methods works satisfactorily for the data sets under consideration herein. In general, performance can be further improved by incorporating prior knowledge about T and/or A if available. Specifically, we have the following two forms of such prior knowledge in mind.

- One or more columns of T may be known in advance given reference profiles obtained from methylation measurements on isolated cell types.
- It is common to have additional knowledge about the cellular composition of the samples the methylation measurements in D are based on. For example, the composition of blood cells is well-studied, and the relative abundance of the underlying major cell types can be narrowed down to intervals.

Both scenarios can be taken advantage of by straightforward modifications of Algorithm 1.

- In case some of the columns of T are known, without loss generality, we may partition $T = [T_0\ \widetilde{T}]$, where T_0 denotes the sub-matrix assumed to be known while $\widetilde{T}$ still needs to be determined. Accordingly, in Algorithm 1 the update of T is confined to $\widetilde{T}$, whereas T_0 is kept fixed over all iterations.

- Lower and upper bounds on the proportions of specific cell types directly translate into identical bounds on the entries of entire rows of A. To give an example, suppose it is known that the proportion of cell type X ranges between 30% and 45% and that of a second cell type Y between 10% and 20%. Unless columns of T are known in advance (see above), problem (3) is invariant to the ordering of the rows of A and we may add the following constraints:

  $$0.3 \leq A_{1j} \leq 0.45, \qquad 0.1 \leq A_{2j} \leq 0.2, \;\; j = 1, \ldots, n.$$

  If a subset of the columns of T is known, one proceeds accordingly: the row indices in A associated with the bound constraints either have to match those corresponding to T_0 or can be chosen freely among the rest, depending on whether prior knowledge about cell type proportions concerns cell types with known respectively unknown methylation profiles.

None of the above modifications make the optimization problem (3) more difficult. Additional bound constraints on the rows of A come into play for optimization problem **optA** in Algorithm 1 and can be handled in a straightforward manner by most solvers of convex quadratic programs. Partial knowledge of T makes optimization even easier.
